# Histone fold domain positioning dictates cotranslational heterodimeric assembly of paralogous TAF12/TAF12L in *Candida albicans*

**DOI:** 10.1016/j.jbc.2026.111239

**Published:** 2026-02-04

**Authors:** Vidhi Bhardwaj, Selene Swanson, Laurence Florens, Michael P. Washburn, Jerry L. Workman, Krishnamurthy Natarajan

**Affiliations:** 1 Laboratory of Eukaryotic Gene Regulation, School of Life Sciences, Jawaharlal Nehru University, New Delhi, India; 2 Stowers Institute for Medical Research, Kansas City, Missouri, USA; 3 Department of Cancer Biology, University of Kansas Medical Center, Kansas City, Kansas, USA

**Keywords:** gene regulation, TAF, TFIID, SAGA, histone fold domain, *Candida albicans*

## Abstract

The fidelity of assembly of multiprotein complexes is essential for the formation of stable and functional protein complexes that are critical for cell growth and survival. In this context, TBP-associated factor (TAF) subunits maintain tight specificity for their integration into TFIID and SAGA complexes. In this work, using affinity purification-coupled mass spectrometry of epitope-tagged TFIID subunits TBP and TAF11, and the SAGA subunit TAF12L we identified components of the *Candida albicans* TFIID and SAGA complexes. Whereas TAF12 is a subunit of TFIID, the paralogous TAF12L is a subunit of the SAGA complex, and we further identified each of the TFIID and SAGA complex subunits with high confidence. We found that the steady-state levels of the histone fold domain containing pairs, TAF12-TAF4 and TAF12L-Ada1 proteins, are mutually dependent on the stable expression of each other. Using RNA immunoprecipitation from polysome-containing extracts, we found that nascent TAF4 and Ada1 proteins interact with TAF12 and TAF12L, respectively, by a cotranslational mechanism in an ordered, sequential mode of assembly. Our results further revealed that the intrinsic position of the histone fold domain within the protein sequence is crucial for determining the sequence and directionality of cotranslational assembly, ensuring both selectivity and stability of the histone fold domain containing heterodimeric proteins in the fungal pathogen *C*. *albicans*.

Assembly of large multisubunit protein complexes requires high fidelity interaction between subunits to avoid nonspecific, deleterious associations for the organism. Nascent protein folding pathways either could be intrinsic to the proteins or mediated by chaperone proteins, and thus heterodimerization of proteins occur posttranslationally by assembly and folding of interacting proteins. Such protein-protein interactions are predominantly driven by specific interacting surfaces in the protein domains but have to contend with specificity challenges especially for paralogous proteins and protein isoforms. An attractive and emerging mechanism for heterodimeric assemblies is by cotranslational assembly, which posits that protein folding and assembly begins even as the polypeptide chains are synthesized on the ribosomes. Indeed, cotranslational assembly has been reported for both eukaryotic and prokaryotic cells (1, 2) and cotranslational assembly provides faithful recognition for interacting subunits rather than by a random collision process.

The type of cotranslational assembly is determined by the way the nascent partners interact. When both polypeptides interact at their nascent stage, it is called a co-co or simultaneous cotranslation (3, 4). The co-co assembly is prevalent in prokaryotic systems for the formation of homodimers or heterodimers (1, 4), such as in the bacterial the assembly of LuxA and LuxB subunits translated from polycistronic mRNA (3). Besides, protein complex assembly also has been shown to take place in a co-post or sequential assembly (2, 5, 6), wherein a fully synthesized protein upon exiting the ribosomes interacts with a nascent polypeptide on or near the ribosome. Recent studies have identified cotranslational interactions that occur through either simultaneous or sequential mechanisms of protein subunit assembly in eukaryotes (3, 6, 7).

In eukaryotes, the transcriptional preinitiation complex (PIC) is composed of the general transcription factors TBP and TFIID besides other GTFs and RNA pol II (8, 9). TFIID is a multisubunit complex composed of TBP and 14 TBP-associated factors (TAFs), and five of these TAFs, TAF5, -6, -9, -10, and −12, are shared in the multifunctional SAGA histone acetyltransferase complex (10, 11, 12). A large body of work led to identification of TAF4/TAF12, TAF6/TAF9, TAF11/13, TAF8/TAF10, and TAF3/TAF10 heterodimeric pairs bearing histone fold domains (HFDs) in the TFIID complex (13, 14, 15). In the SAGA complex too, the TAF or TAF-like protein histone fold pairs are found *viz*., TAF6/TAF9, Ada1/TAF12 and Spt7/TAF10. In multicellular organisms, in particular, several of the TAFs are encoded by paralogous genes that have tissue-specific expression and function (16, 17). Thus, homologous HFD-bearing paralogous proteins offer additional challenges in selectively forming cognate heterodimeric partners.

The human fungal pathogen *Candida albicans* is a critical priority group fungal pathogen (18). We previously showed that *C*. *albicans* genome encodes two TAF12 paralogs, TAF12 and TAF12L that associate with TFIID and SAGA complexes, respectively (19). In this study, we used multidimensional protein identification technology (MudPIT) analysis to identify proteins associated with TAF12-, TAF12L-, and TBP-containing complexes in *C*. *albicans*. Our data show that TAF12 and TAF12L are unique subunits of the TFIID and SAGA complexes, respectively. To understand how the two TAF12 paralogs maintain specificity of association with their HFD pairs *viz*., TAF4 and Ada1, we tested if TAF12 and TAF12L associate with their cognate dimerization partners at the cotranslational stage to impart stability and selectivity. Here, we report that heterodimerization of both TAF12L-Ada1 and TAF12-TAF4 occur through a sequential cotranslational mechanism. Our data also indicate that positioning of the HFD in the interacting partners determine the sequence of cotranslational binding of partners.

## Results

### MudPIT analysis revealed that TAF12 and TAF12L paralogs are unique subunits of TFIID and SAGA in *C*. *albicans*

In a previous study using coimmunoprecipitation assays from cell extracts, we reported that the *C*. *albicans* TAF12 paralogs, TAF12 and TAF12L, interact with TFIID and SAGA complexes, respectively and also showed that TAF4 immunoprecipitated TAF12 whereas Ada1 immunoprecipitated TAF12L (19). To further understand the TAF12 and TAF12L interactions with TFIID and SAGA, we employed MudPIT analysis using immunopurified protein complexes. Toward this end, we affinity purified TFIID complex using *C*. *albicans* strains with endogenous TBP-TAP and TAF11-TAP tagged subunits along with untagged control strain SN87, and subjected it to mass spectrometric analysis, as illustrated in Figure S1. Similarly, to affinity purify SAGA complex, we used a 3xFLAG-tagged TAF12L strain and as an untagged control strain SN87 was used. Subsequently, we carried out affinity purifications using anti-FLAG M2 affinity agarose, protein complexes were eluted, and mass spectrometry analysis was carried out. We analyzed the MudPIT results for proteins showing significant enrichment, based on distributed normalized spectral abundance factor (dNSAF) scores and peptide coverage, for the known TFIID and SAGA subunits as per the genome annotations in the *Candida* Genome Database (20).

**Figure 1:**
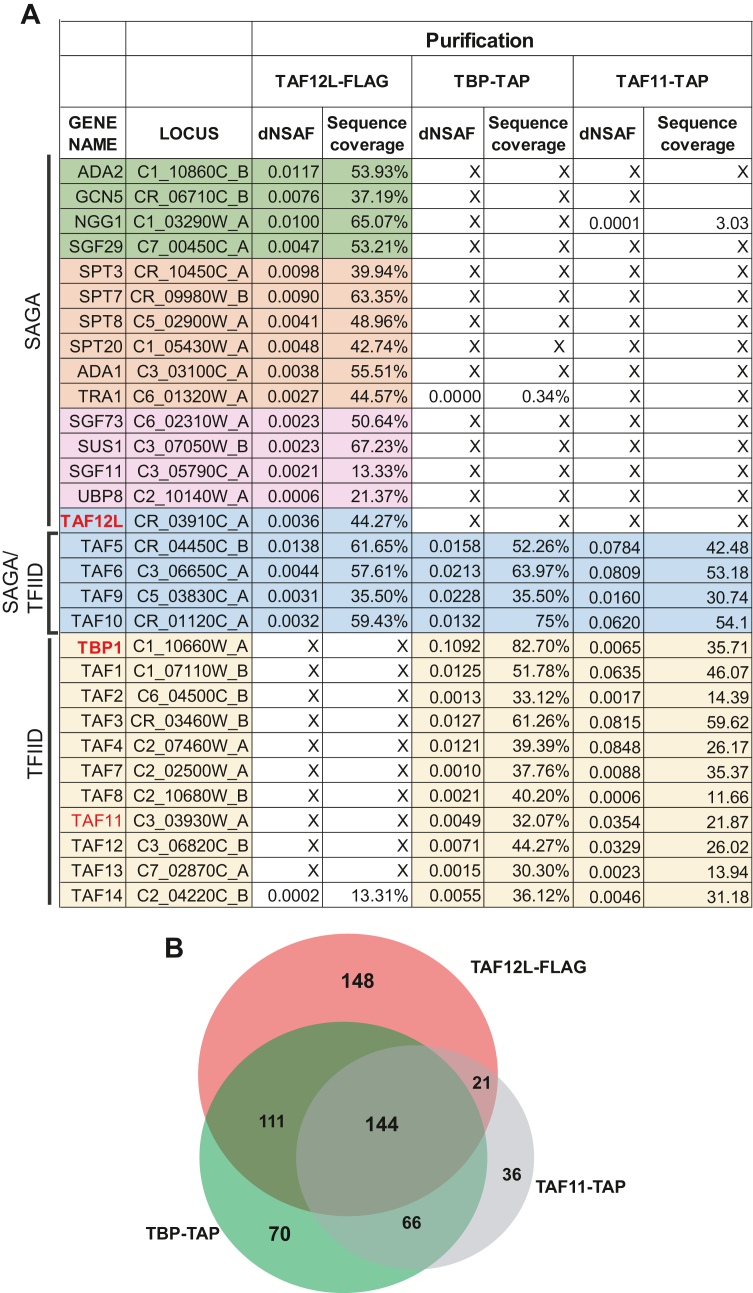
**MudPIT identification of *C*. *albicans* TFIID and SAGA**. *A*, List of TFIID and SAGA subunit proteins identified from MudPIT data. The distributed normalized spectral abundance factor (dNSAF) and percentage sequence coverage for each protein are shown. The homologs of protein subunit names were obtained from *Candida* Genome Database annotation, or were assigned in this study as per homology to the *Saccharomyces cerevisiae* proteins. *B*, *Venn diagram* showing the TFIID and SAGA subunits enriched upon immunopurification-coupled mass spectrometry of TAF12L-FLAG, TBP-TAP, and TAF11-TAP purifications. MudPIT, multidimensional protein identification technology; TAF, TBP-associated factor.

We identified homologs of each of the subunits of the *Saccharomyces cerevisiae* SAGA complex (12, 21) in the TAF12L-FLAG purified sample, including the shared TAFs *viz*., TAF5, -6, -9, and -10 (Fig. 1*A*). However, none of the other TFIID subunits were identified in this preparation (Fig. 1*A*; Table S1). In the TAF11-TAP and TBP-TAP purified samples, homologs of each of the yeast TFIID subunits including TAF12, but not TAF12L, was identified in both these immunopurifications (Fig. 1*A*; Table S1). These results indicate that TAF12L and TAF12 are subunits of the *C*. *albicans* SAGA and TFIID complexes, respectively. Our study also identified several additional proteins that interact with TBP, TAF11, and TAF12L (Tables S1–S2), beyond the bonafide TFIID and SAGA subunits. Thus, unlike in *S*. *cerevisiae*, where TAF12 is shared in both TFIID and SAGA complexes, in *C*. *albicans* paralogous TAF12 and TAF12L show exclusivity for complex association. Furthermore, the overlapping and unique proteins enriched across the three immunopurifications raises the possibility of uncovering novel interactors as well. (Fig. 1*B*, Tables S1–S2).

### Interdependence of TAF12L-ADA1 and TAF12-TAF4 for protein stability

**Figure 2:**
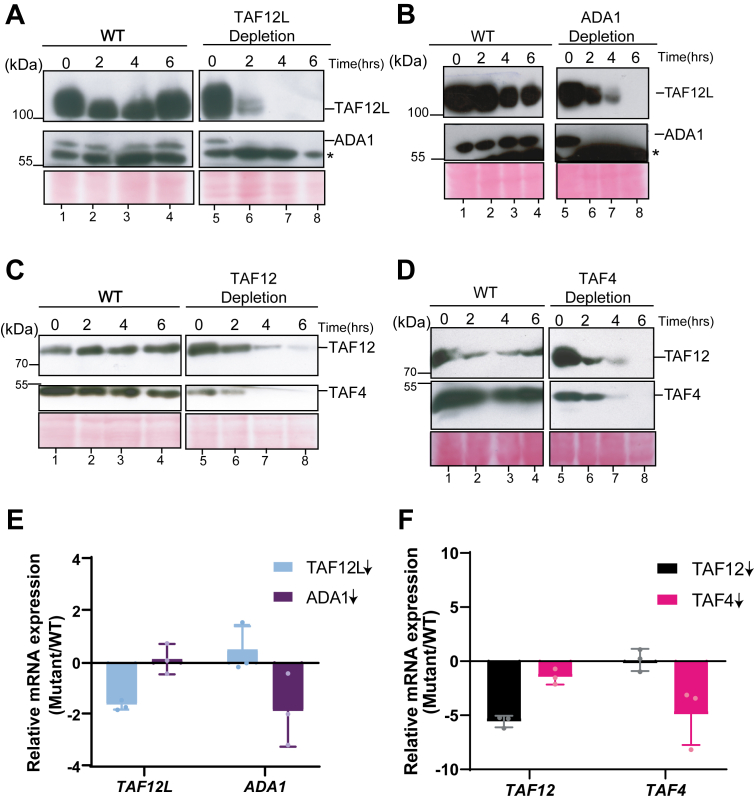
**Protein stability of the TAF12L-Ada1 and TAF12-TAF4 heterodimers is dependent on each partner**. *A–D*, western blot analysis of cell extracts from *A*, ISC11 upon TAF12L (*P*_*MAL2*_-*TAF12L*) depletion. *B*, VBC6 upon Ada1(*P*_*MAL2*_-*ADA1*) depletion, *C*, ISC12 upon TAF12L (*P*_*MAL2*_-*TAF12*) depletion, *D*, VBC4 upon TAF4 (*P*_*MAL2*_-*TAF4*) depletion, or (*A–D*, *Left*) from SN95 (WT) at indicated time points from 0 h to 6 h after shift from YP + maltose (*P*_*MAL2*_ ON) to YP + glucose (*P*_*MAL2*_ shutoff). The lower band in the blot probed with anti-Ada1 is a cross-reacting band marked with an *asterisk* (∗). *E–F*, mRNA levels of indicated transcripts upon *TAF12L*, *ADA1*, *TAF12*, or *TAF4* depletion (marked with *down arrow*). Total RNA was extracted from WT, ISC11, ISC12, VBC4, and VBC6 strains after culturing for 6 h in yeast extract-peptone-dextrose medium at 30 °C. qPCR analysis was carried out, and relative mRNA expression level (mutant/WT) was plotted, normalized to *SCR1* RNA as endogenous control. Error bars indicate ± SD (N = 3). qPCR, quantitative PCR; TAF, TBP-associated factor.

Whereas *C*. *albicans TAF12* is essential for cell growth, *TAF12L*, is not essential but was required to maintain WT yeast cell morphology (19). The human and the budding yeast TAF12, bearing HFDs forms heterodimers with HFD of Ada1 and TAF4 (14, 19, 22). As past studies have shown that interacting subunits become unstable when a dimerization partner is removed (23), or when expressed alone in bacterial cells (24), we tested the impact of removal of TAF12, TAF4, TAF12L, or Ada1 using conditional depletion mutants as described previously (19). The promoter-replaced strains ISC11 (*P*_*MAL2*_*-TAF12L*), VBC6 (*P*_*MAL2*_*-ADA1*), ISC12 (*P*_*MAL2*_*-TAF12*), VBC4 (*P*_*MAL2*_*-TAF4*) and SN95 (WT) were cultured overnight in YP medium containing maltose, diluted into fresh YP medium containing glucose, cells harvested at 0 h, 2 h, 4 h, and 6 h, and protein levels were examined by western blot analysis. The data showed that TAF12L depletion led to reduction in Ada1 protein levels by 2 h (Fig. 2*A*, lane 6), and similarly Ada1 depletion diminished TAF12L protein level by 4 h (Fig. 2*B*, lane 7). In the parental control strain, however, both TAF12L and Ada1 protein levels remain unchanged at all time points (Fig. 2, *A–B*, lanes 1–4). We also examined the effect of TAF12L and Ada1 depletion on TAF12 and TAF4 protein levels. Upon depletion of either TAF12L or Ada1, protein levels of TAF12 and TAF4 remain largely unchanged (Fig. S3, *A*–*B*). Next, we examined the level of TAF4 and TAF12 proteins upon TAF12 (ISC12) or TAF4 (VBC4) depletion in the respective strains. We observed that the TAF4 protein level was undetectable upon TAF12 depletion by 2 h (Fig. 2*C*, lane 7), and reciprocally, TAF12 level was substantially diminished upon TAF4 depletion after 6 h (Fig. 2*D*, lane 8). In parental control strain, the level of both TAF12 and TAF4 remain unchanged (Fig. 2, *C–D*, lanes 1–4). Similarly, TAF12 or TAF4 depletion did not lead to depletion of TAF12L or Ada1 proteins, respectively (Fig. S4, *C–D*). We also tested the growth phenotypes of *TAF4* (VBC4) and *ADA1* (VBC6) depletion mutants and found that *TAF4* is essential but not *ADA1* for cell growth in yeast extract-peptone-dextrose medium (Fig. S2). Furthermore, to examine if the effect of depletion observed at protein levels was due to transcriptional downregulation of their respective mRNAs, we examined *TAF12*, *TAF4*, *TAF12L*, and *ADA1* mRNA levels post 6 h of depletion. We found that the mRNA levels encoding the heterodimerizing partners do not change significantly upon depletion (Fig. 2, *E–F*). Together, these results showed that heterodimerizing partners require each other for protein stability and their individual depletion led to a specific destability of their cognate partner proteins. These data suggest that the heterodimeric proteins could provide chaperoning function for each other to maintain protein stability indicating a likely cotranslational mechanism for subunit recognition to provide specificity and stability to the heterodimers as observed in other cellular systems (25, 26, 27).

### TAF12 paralogs associate with their cognate heterodimeric partners in a cotranslational manner

**Figure 3:**
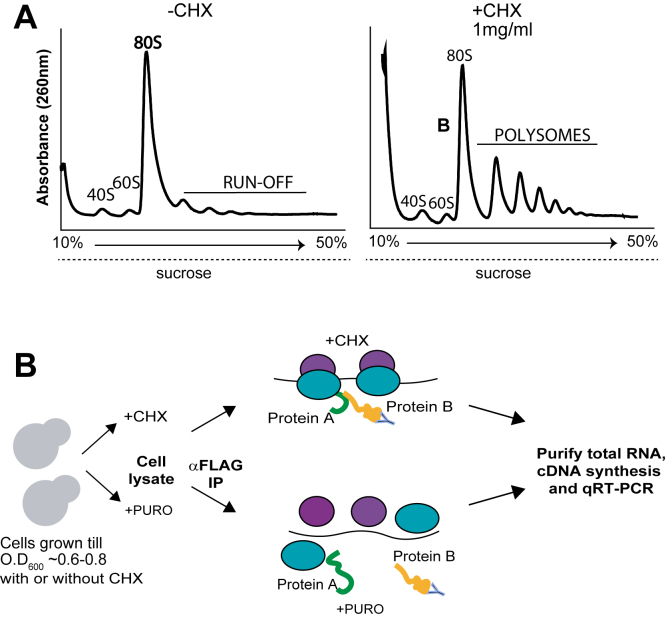
**Schema of RNA immunoprecipitation assay using polysome extracts in *Candida albicans***. *A*, polysome profiles of cell extracts from CHX-treated or untreated *C*. *albicans* cultures fractionated through a 10% to 50% sucrose gradient. *B*, schematic diagram of the RNA immunoprecipitation (RIP) protocol. CHX, cycloheximide

To test whether TAF12 paralogs utilize a cotranslational mechanism for heterodimer recognition, we performed RNA-immunoprecipitation followed by quantitative PCR (qPCR) as previously reported (4, 6, 7, 28). The mRNA enrichment in protein-protein interaction analysis is indicative of a nascent polypeptide association *via* translating ribosomes. Therefore, we prepared polysome-containing cell extracts with the translation elongation inhibitor cycloheximide (CHX) to stall translation and stabilize the RNA-protein interaction *via* ribosome (29). Our initial attempts to cause translational arrest in *C*. *albicans* was unsuccessful using CHX at 100 μg/ml used in other systems, including in the budding yeast (2), due to resistance of *C*. *albicans* ribosomes to CHX (30). Moreover, as previous report obtained polysome profile from *C*. *albicans* treated with 1 mg/ml CHX (31). Therefore, we first tested *C*. *albicans* cell growth at different CHX concentrations from 0.2 mg/ml to 2 mg/ml, and found that *C*. *albicans* growth was not significantly impaired till ∼1 mg/ml CHX (Fig. S4). Next, we prepared polysome extracts, as described in Experimental procedures, from cells treated with CHX (1 mg/ml) or from untreated cells, carried out 10%-50% sucrose density gradient centrifugation, and elution profiles were monitored at A_260_ nm. The polysome profile showed arrested polysomes in CHX-treated cell extracts compared to ribosomal runoff found in the untreated cells (Fig. 3*A*). These results indicated that CHX can be effectively employed at high concentrations for translational arrest to make polysome-containing cell extracts.

**Figure 4:**
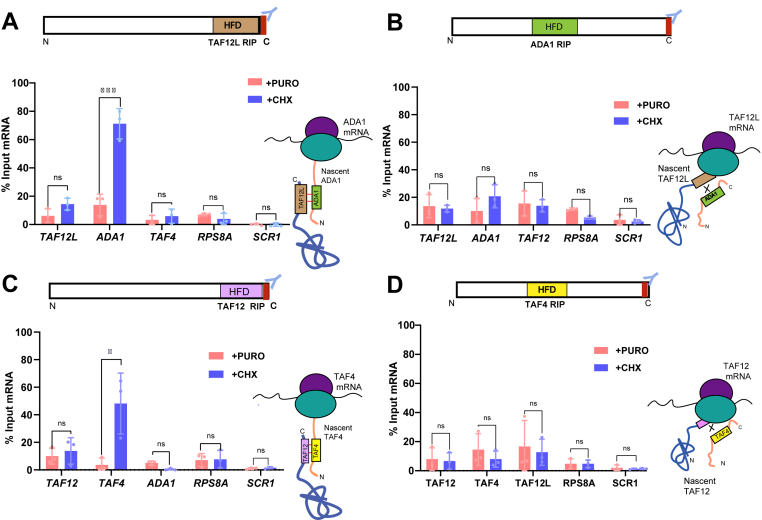
**Cotranslational association of Taf12 paralogs and their heterodimers**: *A*, RIP performed with Taf12L-FLAG-tagged protein as bait from polysome extract treated with either cycloheximide (CHX) or Puromycin (PURO). *B*, RIP performed with the Ada1-FLAG-tagged protein. *C*, RIP with TAF12-FLAG, and *D*, RIP with TAF4-FLAG. The mRNA enrichment was calculated for each mRNA after normalization corresponding input values, separately for puromycin- and cycloheximide-treated samples, and plotted as percent input mRNA. The values were determined from three biological replicates each, and the error bars represent ±SD. The significant difference between CHX and PURO-treated samples for each mRNA was determined by applying a student’s *t* test. *p* value ≤ 0.05 (∗), ≤0.01 (∗∗), ≤0.001 (∗∗∗), and >0.05 (ns). RIP, RNA immunoprecipitation; TAF, TBP-associated factor.

Next, we carried out RNA immunoprecipitation (RIP) assays using polysome-containing cell extracts as schematically depicted in Figure 3*B*. *C*. *albicans* cells were grown in yeast extract-peptone-dextrose medium, and either treated with 1 mg/ml CHX before harvest, or untreated cells as a control. Polysomal cell extracts were prepared from both CHX-treated and untreated cells, and to further ensure complete ribosomal runoff, the untreated control cell extracts were treated with translation inhibitor puromycin that releases nascent peptides from ribosomes. Using anti-FLAG M2 beads, we immunoprecipitated TAF12L-FLAG from both CHX- and puromycin-treated cell extracts. RNA was isolated from the both immunoprecipitated and from input samples and subjected to reverse transcription-linked quantitative PCR analysis. The TAF12L RIP strongly enriched the *ADA1* mRNA in CHX-treated conditions compared to puromycin-treated samples, whereas *TAF4*, *TAF12L*, or *RPS8A* mRNAs or the control *SCR1* RNA did not show any significant enrichment difference between the CHX and puromycin conditions (Fig. 4*A*). These results indicated that TAF12L binds to nascent ribosome-associated Ada1 thereby enriching *ADA1* mRNA (Fig. 4*A*). Conversely, RIP with C terminally tagged Ada1-FLAG as bait showed no enrichment of *TAF12L*, *ADA1*, *TAF12*, control mRNAs *SCR1*, or *RPS8A* in CHX or puromycin-treated conditions (Fig. 4*B*). Taken together, the RIP analysis of both TAF12L and Ada1 proteins indicated that the fully translated TAF12L having exited the ribosome binds to nascent Ada1 polypeptide and, as a result, pulls down ribosome-associated *ADA1* mRNA.

Next, to examine whether TAF12 interacts with TAF4 also in a cotranslational manner, we carried out RIP from TAF12-FLAG containing polysomal extracts as described above. The qPCR analysis of RNA-IP samples from TAF12-FLAG showed strong enrichment of *TAF4* mRNA from CHX-treated, but not from puromycin-treated cell extracts (Fig. 4*C*). Other mRNAs that we tested alongside, *i*.*e*., *ADA1*, *TAF12*, and the control *SCR1* RNA and *RPS8A* mRNA, were not enriched from either of the treated cell extracts (Fig. 4*C*). Interestingly, RIP from C terminally tagged TAF4-FLAG did not enrich *TAF12* mRNA and the other RNAs tested (Fig. 4*D*) indicating that TAF12 interacts cotranslationally with TAF4 but not *vice versa*. Taken together, our results support the sequential cotranslational assembly model for TAF12-TAF4 and TAF12L-Ada1 heterodimerization dependent on the position of the HFD. Thus TAF12 and TAF12L HFD, located near the C-terminal region, facilitates association with nascent TAF4 and Ada1, respectively, bearing HFD toward their N-terminal region, thus enabling an early stage interaction during translation and stability of the interacting partners (26).

## Discussion

In this report, we investigated the composition of protein complexes associated with the TAF12 paralogs from *C*. *albicans*, and showed that CaTAF12 and CaTAF12L are specific subunits of TFIID and SAGA complexes. Our proteomic analyses further identified bonafide subunits of *C*. *albicans* TFIID and SAGA complexes. This work also led to the identification that the two *C*. *albicans* TAF12 paralogs associate with their heterodimerization partners through a cotranslational mechanism, which will be the first example, in our view, of how TAF paralogs could ensure specificity of target protein heteromerization.

Although the coordinated and correct assembly of protein complexes is crucial for cell survival, the posttranslational interaction of partner proteins poses significant challenge due to potential nonspecific interactions in a high concentration cellular environment. This is of particular challenge for proteins with highly similar interaction domains such as those in paralogous proteins. Thus, how the *C*. *albicans* TAF12 and TAF12L proteins that share ∼77% sequence similarity and ∼57% sequence identity between their HFDs (19), maintain selectivity in their heteromeric interactions *viz*., TAF12/TAF4 or TAF12L/Ada1 is of fundamental importance.

The wide array of cotranslationally assembled proteins such as transcription factors, nucleoporins, and cytoskeletal proteins, indicated the extensiveness of cotranslational assembly as a general mechanism in eukaryotes (4, 6, 7, 32, 33). It was previously shown that histone fold domain-mediated dimerization of human TAF8-TAF10 and TAF6- TAF9 (6). Therefore, we tested if the TAF12 and TAF12L paralogs could interact with TAF4 and Ada1, respectively, at cotranslational step by carrying out RIP from translationally arrested polysomal extracts. Our results showed that indeed TAF12L coimmunoprecipitated the *ADA1* mRNA, and TAF12 coimmunoprecipitated the *TAF4* mRNA (Fig. 4, *A* and *C*) in CHX-treated but not in puromycin-treated extracts (Fig. 4, *A* and *C*). In a reciprocal experiment, however, Ada1 and TAF4 proteins could not immunoprecipitate *TAF12L* and *TAF12* mRNAs, respectively (Fig. 4, *B* and *D*).

Because the HFD is located toward the C terminus of the TAF12 paralogs, our results are consistent with the TAF12 and TAF12L interaction TAF4 and Ada1 in a sequential mode of assembly. The antipodal locations of the interacting HFD on these interacting TAFs could give them a spatiotemporal advantage for the association to occur during ongoing translation. Although we have not experimentally shifted the positions of the HFDs to directly test this hypothesis, the observed patterns are consistent with a model in which domain position influences the sequence and directionality of assembly (6). Our assays thus detect pairwise association from the pool of fully synthesized TAF12L and TAF12 proteins with the nascent partners Ada1 and TAF4 to be assembled into SAGA and TFIID complexes. Our western blot data showing the loss of the interacting partners upon depletion of cognate binding partners (Fig. 2, *A*–*D*) also suggests a protective, chaperoning role for the respective interacting partners. These results suggested a quality control mechanism for protein degradation of orphaned subunits in the absence of its binding partner (23, 34), as persistently unbound and unfolded proteins could cause cellular toxicity. In the context of *C*. *albicans*, this mechanism may have broader physiological relevance since the selective degradation of key transcriptional and functional proteins upon exclusion of a binding partner could be exploited as a vulnerability to inhibit fungal growth, as loss of essential subunits could compromise the integrity of transcriptional complexes and thus overall cell survival (35).

Several studies have identified cotranslational assembly of constituent partners to mediate hierarchical assembly for efficient complex formations for large multimeric proteins such as the nucleoporin assembly in *S*. *cerevisiae*, reducing the time at each step which cannot be attained through simple diffusion of interacting proteins (7). Despite the advantage of cotranslational assembly conferring specificity, this may not be an obligatory mechanism, as same proteins could also assemble with same or different partner proteins in a post-translational assembly process. For instance, TAF10 heterodimerization with TAF8 utilized cotranslational assembly in TFIID, but the same TAF10 did not assemble with SAGA-specific SUPT7 through a cotranslational mode (6, 36). Another such example are the proteins involved in nucleoporins, Seh1 interacts cotranslationally with Nup85 but not with Sea-complex (7). Thus what stands out in our study is that both the TAF12 and TAF12L paralogs adopt sequential cotranslational mode of heterodimerization unlike seen in the other examples that involve both sequential and simultanous cotranslational mode of assembly (6, 7, 36).

Although our study established cotranslational assembly as a mechanism mediating subunit recognition of TAF12 paralogs, we hypothesize that additional factors such as paralog-selective chaperones or proteins could confer specificity with a role to spatiotemporally coordinate the expression and localization of the TAF12 paralogs and their respective partners. Identification of such regulators would provide a complete perspective to understand the fidelity and flexibility of transcriptional coactivator assembly. Moreover, understanding the extent of cotranslationally interacting proteins and their regulation becomes increasingly important, especially in identifying key essential transcriptional factors that govern fungal pathogenesis, as targeting these proteins for degradation could become a novel approach to inhibit fungal invasiveness.

## Experimental procedures

Full experimental procedures can be found in the Supporting Information.

## Data availability

Supplemental material containing figures and methods are provided; all strains and plasmids are available upon request. Original mass spectrometry data underlying this manuscript generated at the Stowers Institute can be accessed from the Stowers original data repository at http://www.stowers.org/research/publications/libpb-2562.

## Supporting information

This article contains supporting information (37, 38, 39, 40, 41, 42, 43, 44).

## Conflict of interest

The authors declare that they have no conflicts of interest with the contents of this article.
