# Supplementary material for: Histone fold domain positioning dictates cotranslational heterodimeric assembly of paralogous TAF12/TAF12L in Candida albicans
Source: J Biol Chem. 2026 Feb 4;302(3):111239. doi: 10.1016/j.jbc.2026.111239 (PMC12969390; doi:10.1016/j.jbc.2026.111239)
Supplement: Supplementary Material [file mmc1.pdf]

**Supporting Information**

Bhardwaj et al. *Histone fold domain positioning dictates…*

**Contents**

1. Table S1: List of proteins identified by MudPIT analysis of TBP, TAF11, and TAF12L purifications used for Venn diagram

2. Table S2: Output from Venn diagram containing the list of overlapping and non-overlapping proteins in the three immunopurifications

3. Table S3. List of Strains

4. Table S4. List of Oligonucleotides

5. Experimental procedures

6. Supplemental References

7. Figure S1. Schematic diagram showing MudPIT analysis

8. Figure S2. Growth phenotype analysis of *TAF4*- and *ADA1*- depleted strains

9. Figure S3. Western blot analysis to examine the expression levels upon depletion of TAF12L, TAF12, Ada1 and TAF4

10. Figure S4. Optimization of cycloheximide concentration for *C. albicans* cell growth

**Table S1: List of proteins identified by MudPIT analysis of TBP, TAF11, and TAF12L purifications used for Venn diagram.**

| TBP-TAP | | | TAF12L-FLAG | | | TAF11-TAP | | |
|---|---|---|---|---|---|---|---|---|
| NAME | TBP-TAP dNSAF AVG | | NAME | TAF12L-FLAG dNSAF AVG | Seq Coverage | NAME | TAF11-TAP dNSAF AVG | Seq Coverage |
| TBP1 | 0.109223 | 82.77 | C1_10620W | 0.174283 | 74.74 | TAF4 | 0.084788 | 26.17 |
| HSP70 | 0.102763 | 67.07 | GAR1 | 0.060717 | 48.92 | CR_03460W | 0.081502 | 59.62 |
| C7_00340C | 0.090178 | 58.81 | NOP10 | 0.05025 | 67.8 | TAF60 | 0.080882 | 53.18 |
| RIM1 | 0.04008 | 49.65 | RPL4B | 0.033637 | 46.28 | CR_04450C | 0.078424 | 42.48 |
| C7_01400C | 0.0376 | 66.19 | RPL43A | 0.030106 | 39.13 | TAF145 | 0.063528 | 46.07 |
| C4_04600C | 0.035292 | 59.49 | RPL25 | 0.023901 | 46.48 | TAF10 | 0.062019 | 54.1 |
| TRI1 | 0.034776 | 42.06 | RPL10A | 0.02346 | 43.32 | C2_02500W | 0.057825 | 35.31 |
| C5_03830C | 0.022751 | 35.5 | RPL2 | 0.02273 | 35.43 | C3_03930W | 0.039747 | 31.03 |
| TAF60 | 0.021303 | 63.97 | RPP2A | 0.020047 | 54.63 | TAF12 | 0.035394 | 21.87 |
| HHF22 | 0.018313 | 61.9 | RPP0 | 0.01938 | 44.87 | C5_03830C | 0.03287 | 26.02 |
| SSA2 | 0.018067 | 72.56 | BBC1 | 0.017051 | 58.18 | RPS7A | 0.015967 | 30.74 |
| RPL4B | 0.015993 | 30.3 | ACS1 | 0.016579 | 59.7 | RPP2A | 0.011951 | 31.72 |
| CR_04450C | 0.015847 | 52.26 | RPL8B | 0.014964 | 59.92 | HSP70 | 0.011874 | 31.48 |
| CR_04310C | 0.015739 | 65.64 | RPL3 | 0.014591 | 43.44 | TBP1 | 0.008814 | 35.37 |
| TAF10 | 0.013185 | 75 | RPL39 | 0.014383 | 32.32 | RPL12 | 0.00795 | 26.98 |
| CR_03460W | 0.012739 | 61.26 | CR_04450C | 0.013834 | 61.65 | YDJ1 | 0.006466 | 35.71 |
| TAF145 | 0.012521 | 51.78 | ADA2 | 0.011703 | 53.93 | SIS1 | 0.006218 | 20.61 |
| TAF4 | 0.012122 | 39.39 | RPP1A | 0.011409 | 20.75 | TAF14 | 0.004786 | 36.64 |
| RPP2A | 0.011983 | 47.22 | RPL10 | 0.011348 | 46.36 | TEF1 | 0.004786 | 36.64 |
| C2_02500W | 0.009817 | 37.69 | RPL12 | 0.010403 | 67.88 | PDA1 | 0.004736 | 24.78 |
| RPL8B | 0.007904 | 42.75 | NGG1 | 0.010033 | 65.07 | RPL30 | 0.004551 | 31.18 |
| TAF12 | 0.007108 | 44.27 | CR_10450C | 0.009812 | 39.94 | RPL18 | 0.004293 | 19.21 |

| | | | | | | | | |
|---|---|---|---|---|---|---|---|---|
| RPL12 | 0.006275 | 63.03 | CR_04870C | 0.009616 | 59.28 | C3_04380C | 0.004051 | 27.18 |
| RPL43A | 0.006029 | 19.57 | SPT7 | 0.009014 | 63.35 | C2_05710C | 0.004033 | 28.3 |
| RPL18 | 0.005765 | 22.04 | SSB1 | 0.008018 | 54.81 | RPL10 | 0.003677 | 20.43 |
| TAF14 | 0.005483 | 36.12 | GCN5 | 0.007602 | 37.19 | RPP2B | 0.003393 | 19.05 |
| RPL14 | 0.005363 | 37.4 | NHP2 | 0.007506 | 49.69 | RNR1 | 0.003196 | 17.76 |
| RPL6 | 0.005042 | 51.7 | RPS12 | 0.007093 | 55.24 | TDH3 | 0.003109 | 18.18 |
| C2_07190C | 0.004888 | 41.38 | RPL15A | 0.006406 | 32.84 | SMT3 | 0.003081 | 15.32 |
| C3_03930W | 0.004851 | 32.07 | RPS6A | 0.005951 | 30.08 | C2_03160C | 0.002703 | 22.95 |
| TEF1 | 0.004279 | 31.22 | SSC1 | 0.005599 | 54.94 | KAR2 | 0.002552 | 25.37 |
| TOA2 | 0.004267 | 40.77 | RPL18 | 0.005453 | 30.65 | TUB1 | 0.002515 | 10.78 |
| RPL10 | 0.004034 | 31.82 | RPL23A | 0.005268 | 35.04 | C2_07190C | 0.00249 | 24.27 |
| YDJ1 | 0.004015 | 44.02 | SPT20 | 0.004805 | 42.74 | RPL6 | 0.002489 | 16.59 |
| C1_00710C | 0.003953 | 42.09 | C2_05830C | 0.00479 | 61.3 | RPP1A | 0.002481 | 16.52 |
| HTA2 | 0.003952 | 29.77 | C7_00450C | 0.004711 | 53.21 | SDH12 | 0.002457 | 13.79 |
| C1_04180W | 0.003698 | 11.54 | RPS8A | 0.00445 | 50.49 | RIM1 | 0.002429 | 8.52 |
| RPL10A | 0.003578 | 33.64 | TAF60 | 0.004397 | 57.61 | RPC10 | 0.00242 | 14.15 |
| RPL30 | 0.003488 | 57.55 | RPS3 | 0.004274 | 33.07 | TAF19 | 0.002401 | 11.39 |
| RPL3 | 0.003327 | 36.76 | RPL14 | 0.004169 | 34.35 | C1_00160C | 0.002391 | 12.59 |
| SIS1 | 0.003126 | 45.48 | C5_02900W | 0.004129 | 48.96 | SSB1 | 0.002342 | 27.4 |
| C2_05710C | 0.00311 | 17.76 | RPL24A | 0.004027 | 24.52 | C4_04160W | 0.002332 | 13.94 |
| SMT3 | 0.0029 | 32.35 | RPL32 | 0.00402 | 23.66 | RPL2 | 0.002318 | 13.86 |
| RPL2 | 0.00262 | 19.29 | C3_03100C | 0.003821 | 55.51 | C1_00710C | 0.002137 | 15.5 |
| RPL39 | 0.002615 | 23.23 | SBP1 | 0.003804 | 45.74 | VMA2 | 0.002092 | 20.72 |
| RPL15A | 0.002538 | 21.08 | C2_05710C | 0.003646 | 26.17 | SIK1 | 0.002024 | 14.79 |
| KAR2 | 0.002506 | 35.08 | TAF12L | 0.003615 | 44.27 | SBP1 | 0.00202 | 10.63 |
| SSB1 | 0.002435 | 41.6 | TAF10 | 0.003198 | 59.43 | MRP7 | 0.001904 | 16.48 |
| RPP2B | 0.002332 | 40.93 | C5_03830C | 0.003103 | 35.5 | TSM1 | 0.001837 | 10.16 |

| | | | | | | | | |
|---|---|---|---|---|---|---|---|---|
| C2_10680W | 0.00211 | 20.72 | RPS14B | 0.003103 | 43.94 | GPM1 | 0.001823 | 20.74 |
| RPP1A | 0.002093 | 12.08 | TEF1 | 0.003066 | 38.65 | CMD1 | 0.001819 | 19.86 |
| HTA1 | 0.001961 | 40.2 | HHF22 | 0.002972 | 43.81 | ERG13 | 0.001758 | 20.82 |
| VMA2 | 0.00195 | 14.15 | C1_00180W | 0.002926 | 30.5 | RPS13 | 0.00174 | 14.39 |
| BRF1 | 0.001939 | 39.19 | RPS15 | 0.002885 | 42.25 | C1_00900W | 0.001724 | 19.35 |
| UBI3 | 0.001916 | 28.03 | RPL6 | 0.002881 | 41.48 | RPL8B | 0.001721 | 30.2 |
| RPP0 | 0.001778 | 27.93 | ERG13 | 0.002768 | 37.92 | RPS25B | 0.001706 | 14.41 |
| TIF | 0.00177 | 21.16 | ACT1 | 0.002749 | 55.03 | RPP1B | 0.001699 | 19.21 |
| RPL24A | 0.00167 | 8.29 | RPL28 | 0.002749 | 33.24 | RPL10A | 0.001668 | 7.8 |
| TUB1 | 0.001651 | 33.01 | TRA1 | 0.002716 | 44.57 | SKP1 | 0.001632 | 20.99 |
| RPL27A | 0.001631 | 30.48 | C1_00710C | 0.00265 | 42.09 | KGD2 | 0.001628 | 12.38 |
| RPL35 | 0.001541 | 12.9 | C2_03560C | 0.002632 | 58.73 | TIF | 0.001583 | 14.81 |
| EFT2 | 0.001537 | 31.7 | RPL19A | 0.002566 | 21.05 | NOP5 | 0.001576 | 11.98 |
| RPS18 | 0.00153 | 19.85 | RPL9B | 0.002553 | 43.98 | C1_12280C | 0.001564 | 10.37 |
| RPL28 | 0.001489 | 16.67 | C3_04380C | 0.002477 | 36.51 | RPS26A | 0.001551 | 10.88 |
| TAF19 | 0.001479 | 26.48 | RPL27A | 0.002438 | 43.38 | C4_04390W | 0.001507 | 15.11 |
| RPS1 | 0.001444 | 19.31 | RPS18 | 0.002421 | 36.55 | GAR1 | 0.001491 | 12.4 |
| RPL19A | 0.001362 | 26.85 | RPS23A | 0.002421 | 31.72 | C7_00790W | 0.001474 | 11.64 |
| TSM1 | 0.001295 | 30.3 | C6_02310W | 0.002296 | 50.64 | C1_03370W | 0.001437 | 12.61 |
| C1_00180W | 0.001294 | 21.05 | C3_07050W | 0.002295 | 67.23 | PDC11 | 0.001379 | 14.52 |
| C4_05820W | 0.001261 | 26.17 | RPL30 | 0.002208 | 57.55 | PDB1 | 0.001379 | 16.94 |
| C1_05720W | 0.001227 | 23.68 | RVB1 | 0.002172 | 41.7 | C1_04180W | 0.001379 | 14.65 |
| PIL1 | 0.001201 | 11.4 | RPL13 | 0.002124 | 28.22 | RPL14 | 0.001371 | 16.15 |
| RPS21 | 0.001188 | 33.12 | C3_05790C | 0.002113 | 13.33 | RPS14B | 0.001359 | 13.2 |
| C3_04380C | 0.001174 | 11.5 | CR_04240C | 0.002078 | 41.75 | BMH1 | 0.001357 | 13.76 |
| C1_02330C | 0.001165 | 27.27 | UBI3 | 0.002021 | 32.64 | RPL27A | 0.001353 | 8.18 |
| RPL21A | 0.001156 | 27.39 | CR_04110W | 0.002021 | 49.4 | C1_04180W | 0.001315 | 11.54 |

| | | | | | | | | |
|---|---|---|---|---|---|---|---|---|
| SKP1 | 0.001127 | 23.38 | RPS21B | 0.002018 | 18.39 | C2_00360C | 0.001305 | 9.16 |
| RPL23A | 0.00108 | 19.68 | RPS19A | 0.002018 | 33.79 | RPS12 | 0.001295 | 13.64 |
| RPS8A | 0.001077 | 19.05 | NOP5 | 0.00197 | 31.2 | RPS19A | 0.001295 | 20.08 |
| PDB1 | 0.001073 | 15.75 | RPL35 | 0.001951 | 25 | ENO1 | 0.001257 | 6.62 |
| C1_00590W | 0.001042 | 24.38 | C2_07190C | 0.001906 | 41.38 | C7_02660C | 0.001227 | 6.22 |
| RPL25 | 0.001042 | 20.73 | MRT4 | 0.001866 | 38.26 | RVB1 | 0.001223 | 15.59 |
| PDA1 | 0.001014 | 25.55 | RPL21A | 0.001829 | 25.62 | RPP0 | 0.001196 | 21.68 |
| SIK1 | 0.001003 | 23.79 | C1_11080W | 0.001729 | 45.43 | C4_05630W | 0.001179 | 11.72 |
| RPL5 | 0.000993 | 17.41 | RPS25B | 0.001672 | 21.9 | C7_03000C | 0.001166 | 16.14 |
| MRT4 | 0.000965 | 17.84 | RPS10 | 0.001653 | 41.53 | C1_05720W | 0.001135 | 11.5 |
| C5_01540W | 0.000954 | 18.31 | RPS16A | 0.001648 | 44.37 | HEM1 | 0.00112 | 15.28 |
| RPS20 | 0.000932 | 25.19 | RPS1 | 0.0016 | 34.38 | MGE1 | 0.001096 | 12.5 |
| LAT1 | 0.00093 | 24.22 | RPS24 | 0.001589 | 25.93 | HSP60 | 0.001075 | 10.69 |
| SBP1 | 0.000918 | 21.48 | RPP2B | 0.001581 | 20.72 | ATP2 | 0.001069 | 17.5 |
| HSP104 | 0.000905 | 37.76 | SIK1 | 0.001545 | 43.6 | RPS42 | 0.001064 | 10.37 |
| ERG13 | 0.000902 | 30.12 | TEF2 | 0.001533 | 38.65 | TIF34 | 0.001061 | 7.62 |
| C4_04820C | 0.00086 | 21.3 | HSP70 | 0.001511 | 35.37 | HSP90 | 0.00106 | 7.02 |
| RPL20B | 0.00086 | 24.52 | RPS28B | 0.001456 | 37.31 | C6_04290W | 0.001057 | 10.25 |
| C2_03950W | 0.000837 | 10.92 | RPP1B | 0.001445 | 14.81 | NSP1 | 0.001018 | 9.52 |
| PPH21 | 0.000822 | 14.47 | TUB1 | 0.001437 | 37.95 | HSP21 | 0.000979 | 7.63 |
| RPS24 | 0.000822 | 14.18 | VMA2 | 0.00141 | 47.07 | RPL19A | 0.000977 | 11.14 |
| CR_05150W | 0.000813 | 19.69 | ASC1 | 0.001354 | 38.49 | RPL9B | 0.000967 | 8.91 |
| RPS9B | 0.000783 | 20.62 | TDH3 | 0.001281 | 47.46 | UBI3 | 0.000962 | 8.04 |
| MRPL19 | 0.000776 | 13.18 | CR_04390C | 0.001276 | 20.88 | CHC1 | 0.000953 | 6.69 |
| RPS12 | 0.000776 | 15.7 | RPL17B | 0.001265 | 21.08 | TIF4631 | 0.000905 | 5.29 |
| C2_07680W | 0.000773 | 16.98 | MRP7 | 0.001254 | 36.5 | SSA2 | 0.0009 | 8.95 |
| C1_00160C | 0.00074 | 10.9 | PR26 | 0.001234 | 28.95 | C1_00180W | 0.000895 | 6.28 |

| | | | | | | | | |
|---|---|---|---|---|---|---|---|---|
| RPL13 | 0.000732 | 10.28 | RPS5 | 0.001214 | 13.78 | RPS8A | 0.000886 | 8.29 |
| C1_00900W | 0.000722 | 18.52 | C5_01700W | 0.001207 | 30 | CR_08290W | 0.000876 | 12.23 |
| CR_10350C_B | 0.000718 | 14.29 | C1_04180W | 0.0012 | 19.23 | HHF22 | 0.000868 | 7.84 |
| PTC2 | 0.000698 | 7.41 | ADH1 | 0.00117 | 42.29 | ASC1 | 0.000862 | 24.34 |
| C2_04570W | 0.000693 | 12.59 | C1_00900W | 0.001142 | 23.41 | C7_00340C | 0.000862 | 24.34 |
| RPP1B | 0.000685 | 23.78 | RPL11 | 0.001121 | 24.71 | URA2 | 0.000855 | 6 |
| C2_03560C | 0.000685 | 20.21 | C4_04390W | 0.001101 | 47.58 | C2_04120C | 0.00083 | 8.74 |
| C1_14500C | 0.000672 | 29.82 | C4_03040W | 0.001084 | 44.44 | MRT4 | 0.00082 | 4.8 |
| C4_03090W | 0.000672 | 15.93 | C1_03620C | 0.00105 | 23.08 | CR_06800C | 0.000814 | 12.38 |
| C4_04160W | 0.000656 | 13.75 | HTA2 | 0.001042 | 35.88 | ADH1 | 0.000809 | 9.15 |
| C2_01740C | 0.000654 | 10.89 | TIF | 0.001032 | 31.23 | RPS6A | 0.000792 | 11.64 |
| RNR1 | 0.000649 | 23.41 | C2_07680W | 0.001019 | 20.21 | LAT1 | 0.000772 | 9.48 |
| RVB1 | 0.000646 | 24.27 | RPS20 | 0.000983 | 10.92 | TPI1 | 0.000752 | 6.59 |
| MRPL3 | 0.000641 | 21.1 | C1_05720W | 0.000971 | 18.67 | RVB2 | 0.000743 | 6.52 |
| GFA1 | 0.000622 | 17.1 | BMH1 | 0.00096 | 29.92 | RPS21 | 0.000737 | 14.66 |
| C5_01050C | 0.000616 | 14.81 | RPS9B | 0.000929 | 17.46 | C4_03410W | 0.000733 | 9.71 |
| PET9 | 0.000614 | 18.78 | EFT2 | 0.00088 | 22.57 | RPS1 | 0.000725 | 5.08 |
| CR_00460C | 0.000606 | 5.91 | C2_05410W | 0.000871 | 12.5 | C4_04820C | 0.000717 | 8.6 |
| RPL17B | 0.0006 | 11.27 | YML6 | 0.000842 | 18.35 | RPL3 | 0.000689 | 8.87 |
| PRT1 | 0.0006 | 17.36 | C5_00820W | 0.000827 | 45.23 | SDH2 | 0.000687 | 6.02 |
| GPM1 | 0.000596 | 10.62 | MRPL37 | 0.000813 | 13.33 | MET3 | 0.000687 | 10.04 |
| RFA1 | 0.000593 | 8.67 | GFA1 | 0.000798 | 28.75 | HTA1 | 0.000686 | 16.15 |
| C1_03370W | 0.000588 | 10.26 | IMG2 | 0.000793 | 34.15 | VMA8 | 0.000668 | 6.64 |
| KGD2 | 0.000587 | 15.35 | C4_02260C | 0.000789 | 26.68 | C5_00030W | 0.000663 | 5.81 |
| CR_03120W | 0.000574 | 15.29 | RPS21 | 0.000783 | 39.76 | C2_10680W | 0.000659 | 6.94 |
| C2_04120C | 0.000569 | 12.78 | RVB2 | 0.000783 | 25.9 | DBP5 | 0.00065 | 4.18 |
| RPL32 | 0.000565 | 11.96 | C1_02330C | 0.000768 | 18.9 | C5_00150C | 0.000649 | 6.64 |

| | | | | | | | | |
|---|---|---|---|---|---|---|---|---|
| HET1 | 0.000563 | 8.2 | HSP60 | 0.000758 | 21.55 | C5_04720C | 0.000648 | 22.73 |
| RPS14B | 0.00056 | 15.68 | C5_01540W | 0.000755 | 18.06 | YML6 | 0.00064 | 7.49 |
| BMH1 | 0.00056 | 10.69 | C7_03000C | 0.000731 | 23.75 | MNT1 | 0.000636 | 7.06 |
| GLC7 | 0.00056 | 13.31 | GPM1 | 0.000708 | 20.16 | SUB2 | 0.000636 | 11.66 |
| TDH3 | 0.000552 | 20.83 | RPT6 | 0.000681 | 20.7 | C4_04330C | 0.000633 | 6.85 |
| C5_00030W | 0.00055 | 9.86 | RPL20B | 0.00068 | 25 | PET9 | 0.00063 | 6.81 |
| RPC40 | 0.00055 | 10.88 | RPT5 | 0.00068 | 25.58 | C1_01860W | 0.000629 | 5.51 |
| DBP5 | 0.000548 | 15.53 | CR_10820W_A | 0.000673 | 28.45 | RPL24A | 0.000615 | 3.96 |
| URA2 | 0.000534 | 8.79 | AHP1 | 0.000665 | 50 | SHM2 | 0.000595 | 5.34 |
| PDX1 | 0.000532 | 7.63 | RPL5 | 0.000655 | 17.11 | C7_00070C | 0.000592 | 7.85 |
| MCI4 | 0.000532 | 17.26 | C6_02470W | 0.000645 | 29.92 | MDJ1 | 0.000568 | 3.99 |
| HSP90 | 0.000523 | 13.64 | ATP1 | 0.000643 | 16.67 | C5_02210W | 0.000568 | 5.98 |
| RPS15 | 0.000521 | 34.09 | UBP8 | 0.000636 | 21.37 | SGT2 | 0.000561 | 6.56 |
| C4_03410W | 0.000508 | 14.85 | RPS7A | 0.000629 | 17.2 | HSP104 | 0.000552 | 7.1 |
| CIC1 | 0.000507 | 17.01 | C1_03370W | 0.00062 | 25.12 | PPH21 | 0.000546 | 6.81 |
| ACS2 | 0.000492 | 13.01 | PDC11 | 0.000619 | 23.99 | C4_06680C | 0.000539 | 5.68 |
| DCW1 | 0.000491 | 8.33 | CR_07220C | 0.000619 | 19.52 | RPL4B | 0.000526 | 5.53 |
| RPS13 | 0.00049 | 16.11 | C2_04120C | 0.0006 | 15.16 | CR_00460C | 0.000523 | 6.12 |
| C3_01720C | 0.000485 | 10.38 | TSA1B | 0.000597 | 20.41 | C3_00450C | 0.000497 | 5.23 |
| C1_09620C | 0.00048 | 5.28 | RVS161 | 0.000591 | 18.18 | PRT1 | 0.000476 | 5.67 |
| SHM2 | 0.000472 | 8.63 | C6_03430C | 0.000585 | 22.13 | MP65 | 0.000475 | 5 |
| CR_10830C_A | 0.000468 | 8.46 | C4_03410W | 0.000581 | 16.15 | PFK2 | 0.000472 | 3.53 |
| ASC1 | 0.000467 | 15.28 | HSP90 | 0.000579 | 20.23 | C2_03560C | 0.000471 | 5.23 |
| C5_04990W | 0.000454 | 13.38 | C1_00110W | 0.000578 | 24.44 | RPS9B | 0.000467 | 8.2 |
| MRPL40 | 0.000438 | 13.4 | SSA2 | 0.000577 | 42.95 | LAB5 | 0.000465 | 4.62 |
| C5_00560W | 0.000438 | 12.33 | RPS17B | 0.000569 | 30.66 | C2_03360W | 0.000463 | 3.79 |
| C1_01680C | 0.000435 | 15.24 | CR_10350C_B | 0.000568 | 24.27 | C1_06590C | 0.000452 | 4.23 |

| | | | | | | | | |
|---|---|---|---|---|---|---|---|---|
| C1_01370C | 0.000425 | 7.52 | CR_07080W | 0.000557 | 16.33 | SLK19 | 0.000452 | 5.82 |
| RPL11 | 0.000425 | 7.95 | C5_01050C | 0.000542 | 13.33 | C1_11200W | 0.000452 | 3.28 |
| RPS42 | 0.000423 | 12.69 | C1_12610W | 0.000532 | 16.82 | MRPL3 | 0.000452 | 5.03 |
| ENO1 | 0.00042 | 9.66 | RPS42 | 0.000521 | 29.39 | RPL15A | 0.000443 | 4.92 |
| SDH12 | 0.000404 | 9.74 | C3_06970W | 0.000515 | 13.37 | CAM1 | 0.00044 | 2.06 |
| C4_06730C | 0.000404 | 10 | C5_00030W | 0.000508 | 13.01 | CDC48 | 0.000432 | 7.32 |
| C6_03380W | 0.000402 | 7.59 | CDC19 | 0.000503 | 30.16 | LYS22 | 0.000429 | 5.37 |
| C7_01210C | 0.000399 | 14.2 | RNR1 | 0.000502 | 18.62 | NOG1 | 0.000429 | 2.34 |
| RPS7A | 0.000398 | 10.43 | RPT4 | 0.000501 | 17.99 | C2_04570W | 0.000423 | 4.95 |
| HSP21 | 0.000391 | 10.65 | C5_04910W | 0.000494 | 18.14 | C6_02690C | 0.000419 | 5.88 |
| C4_05900C | 0.000377 | 9.47 | RPS26A | 0.000492 | 12.61 | C1_12610W | 0.000415 | 5.1 |
| RPL7 | 0.000376 | 8.82 | ENO1 | 0.000488 | 16.36 | NUP49 | 0.000414 | 4.12 |
| C5_02380W | 0.000372 | 18.39 | C5_02660C | 0.000466 | 20.6 | ACC1 | 0.00041 | 3.36 |
| MET10 | 0.000372 | 8.05 | RSM22 | 0.000464 | 12.02 | C6_00290W | 0.000401 | 5.63 |
| RVB2 | 0.000371 | 11.45 | C2_03950W | 0.000442 | 20 | C2_02170W | 0.0004 | 5.62 |
| PWP1 | 0.000362 | 3.82 | RPT1 | 0.000439 | 15.32 | C1_05270C | 0.000398 | 5.59 |
| CR_07320C | 0.000359 | 16.14 | CR_00460C | 0.000426 | 8.2 | C1_10620W | 0.000389 | 6.82 |
| MAK16 | 0.000352 | 6.71 | RPN13 | 0.000422 | 19.49 | CR_07080W | 0.000381 | 2.23 |
| NOP1 | 0.000351 | 6.99 | C1_05270C | 0.000408 | 25.94 | LPD1 | 0.000376 | 5.81 |
| C7_00790W | 0.000348 | 13.59 | SAM2 | 0.000405 | 18.44 | CDC53 | 0.000375 | 2.92 |
| SRP40 | 0.000346 | 6.12 | YDJ1 | 0.000397 | 22.14 | CDC19 | 0.000373 | 3.71 |
| GSP1 | 0.000346 | 15.59 | ADE4 | 0.000397 | 28.84 | C3_05880C | 0.000358 | 4.6 |
| CHC1 | 0.000334 | 5.29 | RPS13 | 0.000388 | 19.21 | MTS1 | 0.000357 | 2.71 |
| KGD1 | 0.000334 | 12.76 | SMT3 | 0.000382 | 21.57 | RTG3 | 0.000349 | 9.39 |
| C2_05410W | 0.00033 | 5.76 | SRB1 | 0.000377 | 9.67 | C3_03410C | 0.000348 | 2.65 |
| CR_10740W_A | 0.000329 | 10.05 | C4_05630W | 0.000368 | 16.04 | CCR4 | 0.000341 | 4.39 |
| C2_07370W | 0.000322 | 8.23 | PDB1 | 0.00036 | 18.47 | HEM14 | 0.000339 | 2.38 |

| | | | | | | | | |
|---|---|---|---|---|---|---|---|---|
| C1_11200W | 0.000321 | 7.43 | C2_05160C | 0.000351 | 14.69 | SSZ1 | 0.000335 | 3.73 |
| C1_12280C | 0.000319 | 13.05 | C5_04630W | 0.000348 | 16.96 | C3_00850C | 0.000333 | 3.31 |
| RPS6A | 0.000313 | 6.8 | MRPL40 | 0.000346 | 21.3 | PDI1 | 0.000329 | 4.42 |
| C5_04910W | 0.000312 | 8.25 | C2_01740C | 0.000345 | 10.62 | EFT2 | 0.000327 | 4.49 |
| RPS26A | 0.000311 | 8.23 | ACS2 | 0.000342 | 11.98 | MPP10 | 0.000326 | 3.56 |
| DPM1 | 0.000309 | 4.7 | C1_12280C | 0.000336 | 11.64 | NOT4 | 0.000322 | 3.01 |
| MGE1 | 0.000306 | 9.11 | RPT2 | 0.000336 | 15.52 | CR_00490W | 0.000317 | 3.33 |
| PRX1 | 0.000304 | 6.54 | ACH1 | 0.000335 | 8.4 | PES1 | 0.000314 | 1.96 |
| CR_07080W | 0.000302 | 10.9 | TRP99 | 0.000318 | 7.07 | C4_03290W | 0.000306 | 2.33 |
| LPD1 | 0.000301 | 14.16 | PFK26 | 0.000317 | 20.92 | C5_02380W | 0.000305 | 5.34 |
| ARO8 | 0.000301 | 12.5 | KAR2 | 0.000312 | 12.95 | AAF1 | 0.000302 | 3 |
| IMG2 | 0.000301 | 4.44 | CR_07510W | 0.000312 | 8.8 | MIR1 | 0.000297 | 3.3 |
| NSA2 | 0.000283 | 6.98 | PRE8 | 0.000311 | 6.37 | PIL1 | 0.000292 | 6.14 |
| SDH2 | 0.000281 | 9.88 | RHR2 | 0.000307 | 10.24 | C2_05300C | 0.000291 | 3.41 |
| NOP15 | 0.000277 | 5.17 | C1_00160C | 0.000293 | 14 | LSP1 | 0.000286 | 3.85 |
| RFA2 | 0.000272 | 7.2 | NOG2 | 0.000293 | 12.2 | C3_02350W | 0.000286 | 1.78 |
| MGM101 | 0.000269 | 6.75 | C3_01720C | 0.000288 | 10.73 | DED1 | 0.000279 | 2.78 |
| HEM1 | 0.000262 | 7.56 | C3_06760W | 0.000284 | 4.36 | ILV2 | 0.000278 | 5.52 |
| HSP60 | 0.000261 | 4.6 | PGK1 | 0.000281 | 11.51 | DRG1 | 0.000278 | 5.54 |
| ZUO1 | 0.00026 | 7.02 | ALI1 | 0.00028 | 10.04 | IDH2 | 0.000272 | 5.41 |
| NSP1 | 0.000258 | 4.94 | C1_11880W | 0.000279 | 7.14 | C4_02260C | 0.00027 | 5.36 |
| RPT5 | 0.000258 | 15.92 | C2_04570W | 0.000274 | 9.37 | C5_03550W | 0.000256 | 2.85 |
| SUB2 | 0.000256 | 8.76 | GCD2 | 0.000273 | 8.6 | FLO8 | 0.000254 | 1.93 |
| RPD31 | 0.000256 | 8.35 | UBC4 | 0.000265 | 18.37 | C4_00420C | 0.00025 | 2.78 |
| C6_00550W | 0.000246 | 10.57 | PRT1 | 0.000264 | 9.88 | C2_04370W | 0.000232 | 3.8 |
| C4_06680C | 0.000245 | 5.36 | C4_06210C | 0.00026 | 12.55 | PGK1 | 0.000232 | 4.88 |
| C6_02350C | 0.000242 | 4.18 | TKL1 | 0.000259 | 6.06 | PDX1 | 0.00023 | 5.39 |

| | | | | | | | | |
|---|---|---|---|---|---|---|---|---|
| SEC24 | 0.000239 | 7.42 | RPN1 | 0.000255 | 11.28 | PIN4 | 0.000216 | 1.39 |
| C1_03280W | 0.000239 | 4.87 | C2_02270C | 0.000252 | 9.03 | C1_08180C | 0.000216 | 2.02 |
| CR_04170W | 0.000239 | 11.4 | RNH1 | 0.000251 | 15.45 | UTP9 | 0.000211 | 3.82 |
| YTM1 | 0.000238 | 4.36 | LAT1 | 0.000245 | 9.64 | ROM2 | 0.000209 | 3.41 |
| SNF12 | 0.000237 | 3.37 | NIP1 | 0.000245 | 9.27 | SER33 | 0.000205 | 3.6 |
| C1_00930C | 0.000236 | 10.42 | ARO8 | 0.000238 | 13.85 | SNF12 | 0.000205 | 4.32 |
| C4_05630W | 0.000233 | 3.28 | TPI1 | 0.000236 | 8.87 | ARO8 | 0.000199 | 1.97 |
| CDC28 | 0.000233 | 7.21 | GSY1 | 0.000236 | 19.52 | KGD1 | 0.000195 | 1.21 |
| LSP1 | 0.000233 | 9.05 | ALD5 | 0.000235 | 14.43 | CAP1 | 0.000192 | 3.6 |
| C7_03000C | 0.000231 | 7.16 | C1_05650W | 0.000232 | 8.73 | C3_00130C | 0.000186 | 1.16 |
| C6_02690C | 0.00023 | 7.8 | C7_03830C | 0.000232 | 8.33 | C1_12030W | 0.000185 | 6.48 |
| ACC1 | 0.000228 | 7.97 | C1_00590W | 0.000229 | 14.08 | YWP1 | 0.000182 | 2.99 |
| C4_00420C | 0.000228 | 4.08 | C1_00040W | 0.000229 | 12.94 | ADE4 | 0.000174 | 3.67 |
| MDJ1 | 0.000227 | 3.1 | C4_04820C | 0.000227 | 8.14 | SRV2 | 0.000172 | 2.01 |
| C5_03980W | 0.000224 | 6.79 | C7_03660C | 0.000226 | 9.84 | MLS1 | 0.000171 | 3.01 |
| GCD2 | 0.000222 | 5.48 | C5_03410C | 0.000225 | 5.2 | CR_02610C | 0.000163 | 2.1 |
| ILV2 | 0.000217 | 3.72 | YST1 | 0.000224 | 17.24 | UBR1 | 0.000161 | 3.2 |
| NOP5 | 0.000215 | 7.08 | VMA6 | 0.000224 | 14.37 | KRE30 | 0.00016 | 3.19 |
| AHA1 | 0.000214 | 7.46 | TAF14 | 0.000222 | 13.31 | C1_01590C | 0.000158 | 2.59 |
| C2_01390W | 0.000213 | 6.66 | C1_01580W | 0.000222 | 6.82 | HIS7 | 0.000157 | 3.12 |
| ADH1 | 0.000211 | 5.66 | LYS22 | 0.000218 | 12.23 | PGA63 | 0.000155 | 2.9 |
| MET3 | 0.00021 | 4.73 | C5_00150C | 0.000216 | 13.63 | SSD1 | 0.000148 | 5.21 |
| YWP1 | 0.000208 | 3.47 | IDH1 | 0.000214 | 11.54 | C6_00170C | 0.000144 | 2.24 |
| FBA1 | 0.000206 | 6.25 | CCT7 | 0.000213 | 12.39 | TKL1 | 0.00014 | 4.93 |
| TIF4631 | 0.000205 | 7.92 | PGI1 | 0.000213 | 5.82 | ACS2 | 0.000139 | 1.05 |
| C1_05630C | 0.0002 | 4.71 | NOG1 | 0.000213 | 6.72 | PAN1 | 0.000136 | 3.19 |
| TCP1 | 0.0002 | 7.27 | RPN12 | 0.000211 | 11.91 | TRP5 | 0.000135 | 2.69 |

| | | | | | | | | |
|---|---|---|---|---|---|---|---|---|
| GAR1 | 0.000199 | 5.94 | HSP21 | 0.000206 | 5.29 | C1_00930C | 0.000134 | 1.57 |
| RPG1A | 0.000199 | 4.97 | SAR1 | 0.000205 | 10.53 | UTP13 | 0.00013 | 3.8 |
| C7_02960C_A | 0.000197 | 6.8 | CEF3 | 0.000204 | 7.14 | NAN1 | 0.000126 | 5.18 |
| PDC11 | 0.000196 | 9.22 | C1_01600W | 0.0002 | 10.24 | C1_04290C | 0.000126 | 2.95 |
| RPL9B | 0.000194 | 10.85 | RPL7 | 0.000198 | 10.85 | NIP1 | 0.000122 | 2.71 |
| VMA5 | 0.000191 | 8.09 | MRPL3 | 0.000193 | 11.39 | RPG1A | 0.000122 | 1.36 |
| MRP7 | 0.00019 | 12.31 | THS1 | 0.000193 | 11.05 | SEC24 | 0.000121 | 1.56 |
| GPD1 | 0.000184 | 3.26 | PIL1 | 0.00019 | 6.82 | SEC27 | 0.000107 | 1.5 |
| C5_00260W | 0.000183 | 3.14 | CR_07320C | 0.000189 | 9.39 | C1_07340W | 0.000107 | 4.59 |
| NSA1 | 0.000182 | 6.83 | RNR21 | 0.000189 | 10.17 | C1_00960C | 0.000102 | 2.98 |
| AAF1 | 0.000181 | 3.19 | PDX1 | 0.000187 | 11.51 | GLT1 | 0.000099 | 1.27 |
| PGK1 | 0.000177 | 4.46 | HIS7 | 0.000187 | 6.06 | SLA2 | 0.000098 | 1.6 |
| CR_10490W_A | 0.000177 | 3.51 | GLN3 | 0.000186 | 20.67 | SRO77 | 0.000092 | 1.83 |
| LYS22 | 0.000177 | 5.42 | NOP1 | 0.000185 | 5.38 | C1_05630C | 0.000092 | 1.72 |
| C5_01700W | 0.000176 | 6.49 | C2_09660W | 0.000179 | 5.5 | C6_03460W | 0.000091 | 3.62 |
| C1_03790C | 0.000174 | 6.49 | C1_14500C | 0.000177 | 6.36 | RPA135 | 0.000084 | 1.28 |
| ABP1 | 0.000171 | 11.83 | UTP18 | 0.000177 | 8.7 | RGA2 | 0.000082 | 1.15 |
| SSC1 | 0.000171 | 4.09 | C7_03840W | 0.000177 | 6.04 | C1_06550W | 0.00008 | 0.8 |
| C2_09660W | 0.00017 | 4 | UTP9 | 0.000175 | 9.89 | NGG1 | 0.00008 | 2.16 |
| C6_00170C | 0.000169 | 7.41 | C1_10470W | 0.000174 | 12.5 | SPT6 | 0.000079 | 3.07 |
| C1_12610W | 0.000168 | 6.28 | CLC1 | 0.000173 | 7.56 | ECM17 | 0.000077 | 1.8 |
| PRO2 | 0.000166 | 3.87 | FAS1 | 0.000172 | 6.53 | MLP1 | 0.000074 | 1.89 |
| DED1 | 0.000165 | 3.08 | SER33 | 0.000169 | 11.27 | FAS1 | 0.000073 | 2.21 |
| NUP49 | 0.000165 | 3.97 | RPD31 | 0.000169 | 9.72 | C7_00570W | 0.000073 | 1.46 |
| TKL1 | 0.000164 | 5.69 | C6_02880W | 0.000168 | 4.74 | C1_11860W | 0.00007 | 1.56 |
| RPC53 | 0.000164 | 2.96 | URA2 | 0.000167 | 8.71 | C1_03290W | 0.000068 | 3.03 |

| | | | | | | | | |
|---|---|---|---|---|---|---|---|---|
| C2_02170W | 0.000161 | 6.37 | FBA1 | 0.000163 | 11.98 | C7_04300W | 0.000061 | 3.03 |
| RPD3 | 0.000154 | 3.6 | CIC1 | 0.00016 | 4.66 | C2_06170C | 0.00006 | 1 |
| PHO23 | 0.000154 | 5.04 | C6_03380W | 0.000159 | 4.35 | C4_01060W | 0.000047 | 0.77 |
| C1_10620W | 0.000154 | 6.7 | C3_00450C | 0.000159 | 8.6 | C5_00190C | 0.000042 | 0.76 |
| C6_02370C | 0.000153 | 10.48 | ZCF29 | 0.000159 | 8.42 | | | |
| C1_09040C | 0.000152 | 5.16 | PMM1 | 0.000155 | 5.56 | | | |
| DIP2 | 0.000151 | 4.78 | UTP5 | 0.000153 | 8.81 | | | |
| VID27 | 0.000149 | 4.78 | SDH12 | 0.000152 | 7.33 | | | |
| CDC19 | 0.000147 | 7.34 | NSA2 | 0.000149 | 5.36 | | | |
| RHR2 | 0.000146 | 4.71 | C2_07220W | 0.000148 | 16.67 | | | |
| C3_05880C | 0.000145 | 5 | NOP15 | 0.000146 | 4.87 | | | |
| FUN12 | 0.000145 | 3.59 | VMA8 | 0.000146 | 7.49 | | | |
| C3_07460W | 0.000144 | 5.95 | C3_04810C | 0.000146 | 7.87 | | | |
| RTG3 | 0.000142 | 3.79 | CR_02420W | 0.000145 | 5.95 | | | |
| C3_00130C | 0.000141 | 2.66 | RPN2 | 0.000143 | 7.14 | | | |
| RVS161 | 0.00014 | 3.32 | CCJ1 | 0.000142 | 7.3 | | | |
| ADE4 | 0.000137 | 3.71 | MRPL10 | 0.000138 | 3.89 | | | |
| C5_04720C | 0.000136 | 3.75 | MYO2 | 0.000137 | 6.73 | | | |
| C3_00850C | 0.000136 | 3.34 | SUB2 | 0.000135 | 11.78 | | | |
| ATP1 | 0.000135 | 2.7 | DPS1-1 | 0.000135 | 8.3 | | | |
| CCT7 | 0.000135 | 2.07 | RAS1 | 0.000135 | 4.14 | | | |
| RPN13 | 0.000133 | 7.19 | PES1 | 0.000133 | 5.79 | | | |
| ALI1 | 0.000133 | 3.67 | CR_00490W | 0.000133 | 4.78 | | | |
| CTA7 | 0.000133 | 2.15 | UTP4 | 0.000131 | 10.09 | | | |
| RAD52 | 0.000131 | 2.38 | PET9 | 0.00013 | 7.97 | | | |
| RET2 | 0.000129 | 14.57 | C3_06700C | 0.000127 | 6.85 | | | |
| C1_13320C | 0.000129 | 3.73 | RPG1A | 0.000126 | 9.66 | | | |

| | | | | | | | | |
|---|---|---|---|---|---|---|---|---|
| CR_02610C | 0.000128 | 2.85 | YTM1 | 0.000126 | 7.2 | | | |
| CSI2 | 0.000127 | 4.04 | SEC27 | 0.000125 | 8.84 | | | |
| NIP1 | 0.000127 | 3.27 | C5_00080C | 0.000125 | 10.61 | | | |
| PES1 | 0.000126 | 4.76 | PFK2 | 0.000124 | 5.07 | | | |
| MIR1 | 0.00012 | 7.95 | CR_10830C_A | 0.000123 | 11.39 | | | |
| SDS24 | 0.000118 | 7.58 | C2_07290W | 0.000123 | 7.23 | | | |
| RPN1 | 0.000112 | 4.78 | UTP13 | 0.000122 | 6.38 | | | |
| C5_02660C | 0.00011 | 4.29 | CR_03120W | 0.000121 | 4.97 | | | |
| C1_10470W | 0.00011 | 2.38 | HXK2 | 0.000121 | 5.99 | | | |
| FAS1 | 0.000109 | 3.28 | C5_04990W | 0.00012 | 11.04 | | | |
| MSI3 | 0.000106 | 4.33 | C3_07420W | 0.000119 | 5.79 | | | |
| CR_04240C | 0.000106 | 5.02 | CDC48 | 0.000118 | 5.45 | | | |
| TRP5 | 0.000105 | 2.04 | C3_02350W | 0.000117 | 8.55 | | | |
| C3_01850W | 0.000105 | 3.55 | RPA135 | 0.000117 | 4.03 | | | |
| CDC10 | 0.000104 | 4.89 | MSS116 | 0.000117 | 4.64 | | | |
| GZF3 | 0.000104 | 2.44 | RPC40 | 0.000116 | 5.06 | | | |
| SRO77 | 0.000103 | 9.38 | ATP2 | 0.000116 | 6.75 | | | |
| ILS1 | 0.000102 | 9.22 | MRPS9 | 0.000116 | 6.25 | | | |
| IDH2 | 0.0001 | 7.88 | CSH1 | 0.000116 | 5.64 | | | |
| UTP4 | 0.0001 | 1.6 | ILV2 | 0.000114 | 5.56 | | | |
| ACT1 | 0.000098 | 5.45 | GRP2 | 0.000114 | 15.84 | | | |
| ARP7 | 0.000098 | 5.54 | ARC35 | 0.000113 | 5.49 | | | |
| HOG1 | 0.000098 | 2.87 | NMD3 | 0.000113 | 5.19 | | | |
| C5_03000C | 0.000097 | 6.45 | C5_00790C | 0.000112 | 4.01 | | | |
| SAM2 | 0.000096 | 6.27 | C3_01850W | 0.000111 | 3.42 | | | |
| CR_05550C | 0.000096 | 5.36 | CHA1 | 0.000111 | 10.2 | | | |
| C2_03360W | 0.000095 | 3.24 | CDC10 | 0.000109 | 5.04 | | | |

| | | | | | | | | |
|---|---|---|---|---|---|---|---|---|
| C1_06590C | 0.000093 | 11.3 | KRR1 | 0.000109 | 3.63 | | | |
| SLK19 | 0.000093 | 2.28 | CHC1 | 0.000106 | 5.18 | | | |
| FLO8 | 0.000093 | 1.54 | TCP1 | 0.000106 | 5.42 | | | |
| SEC18 | 0.000093 | 4.56 | DRG1 | 0.000106 | 4.08 | | | |
| RDH54 | 0.000093 | 2.71 | LYS21 | 0.000106 | 10.05 | | | |
| CDC48 | 0.00009 | 3.64 | AGO1 | 0.000106 | 5.78 | | | |
| C2_04370W | 0.00009 | 2.39 | C6_04100W | 0.000106 | 5.15 | | | |
| CAM1 | 0.00009 | 2.88 | GCN1 | 0.000105 | 4.8 | | | |
| PR26 | 0.00009 | 2.76 | C5_04640C | 0.000104 | 8.52 | | | |
| CR_08290W | 0.000089 | 4.61 | ERG6 | 0.000104 | 5.32 | | | |
| SMC1 | 0.000089 | 4.04 | ARP7 | 0.000103 | 5.29 | | | |
| RPT4 | 0.000086 | 4.5 | HOG1 | 0.000103 | 4.24 | | | |
| DNM1 | 0.000085 | 2.12 | HET1 | 0.000099 | 10.15 | | | |
| C1_02240W | 0.000085 | 4.26 | RNR22 | 0.000099 | 4.82 | | | |
| PMA1 | 0.000083 | 1.84 | PDA1 | 0.000097 | 4.74 | | | |
| CR_03220C | 0.000083 | 5.97 | C1_00930C | 0.000097 | 4.82 | | | |
| MCM2 | 0.000082 | 2.51 | C5_00260W | 0.000097 | 3.71 | | | |
| CDC54 | 0.000081 | 1.8 | NSA1 | 0.000096 | 2.96 | | | |
| SER33 | 0.00008 | 4.55 | LMO1 | 0.000095 | 5.54 | | | |
| SEC27 | 0.000079 | 2.42 | GLT1 | 0.000092 | 4.84 | | | |
| FAS2 | 0.000079 | 1.64 | TIF4631 | 0.00009 | 2.12 | | | |
| SEC21 | 0.000079 | 4.65 | HSP104 | 0.000087 | 3.45 | | | |
| PFK2 | 0.000078 | 1.01 | SWD1 | 0.000087 | 4.92 | | | |
| SEC26 | 0.000078 | 1.94 | SES1 | 0.000085 | 6.33 | | | |
| ECM17 | 0.000077 | 4.63 | SPT6 | 0.000084 | 3.5 | | | |
| C2_04620W | 0.000076 | 4.62 | SNF12 | 0.000083 | 7.25 | | | |
| PNG2 | 0.000075 | 2.67 | C1_12240C | 0.000083 | 3.4 | | | |

| | | | | | | | | |
|---|---|---|---|---|---|---|---|---|
| PFK1 | 0.000075 | 4.8 | MIS11 | 0.000082 | 3.91 | | | |
| ATP2 | 0.000073 | 0.97 | LPD1 | 0.000079 | 10.39 | | | |
| MTS1 | 0.000072 | 1.71 | C6_03440W | 0.000076 | 4.43 | | | |
| C1_00060W | 0.000072 | 2.19 | ARO9 | 0.000075 | 6.88 | | | |
| C2_06980W | 0.000072 | 2.23 | AIP2 | 0.000074 | 3.8 | | | |
| CR_10470C_B | 0.00007 | 3.36 | HOM3 | 0.000072 | 3.13 | | | |
| SLA2 | 0.00007 | 1.22 | PBS2 | 0.000072 | 2.94 | | | |
| C4_06210C | 0.00007 | 4.06 | NAN1 | 0.00007 | 3.93 | | | |
| CEF3 | 0.00007 | 6.48 | GAD1 | 0.000069 | 4.23 | | | |
| MLS1 | 0.000067 | 1.6 | HAS1 | 0.000069 | 3.54 | | | |
| UTP18 | 0.000067 | 1.59 | CSI2 | 0.000067 | 3.42 | | | |
| RPA190 | 0.000067 | 2.14 | GGA2 | 0.000067 | 2.91 | | | |
| PGI1 | 0.000067 | 3.7 | PWP1 | 0.000064 | 3.59 | | | |
| PDI1 | 0.000066 | 1.16 | C6_02350C | 0.000064 | 3.1 | | | |
| C2_02540W | 0.000064 | 3.27 | APE2 | 0.000063 | 3.79 | | | |
| ELF1 | 0.000062 | 3.81 | PGA63 | 0.000062 | 4.35 | | | |
| KRE30 | 0.000061 | 1.32 | C2_05300C | 0.000062 | 5.41 | | | |
| HIS7 | 0.000059 | 2.23 | SEC26 | 0.000061 | 3.15 | | | |
| C6_04290W | 0.000059 | 2.98 | ACC1 | 0.00006 | 3.43 | | | |
| C3_05360C | 0.000059 | 8.31 | C1_00640C | 0.00006 | 2.77 | | | |
| TOM1 | 0.000056 | 2.34 | C2_06200C | 0.00006 | 1.83 | | | |
| C3_02350W | 0.000055 | 3.31 | GLC7 | 0.000059 | 4.24 | | | |
| UTP8 | 0.000053 | 7.23 | ROB1 | 0.000059 | 2.12 | | | |
| GLT1 | 0.000052 | 3.42 | DED1 | 0.000058 | 5.06 | | | |
| C4_07060W | 0.000051 | 2.1 | RGD1 | 0.000058 | 2.95 | | | |
| CDC53 | 0.000049 | 3.2 | VPS1 | 0.000056 | 2.45 | | | |
| CR_03200C | 0.000047 | 2.92 | PPH21 | 0.000054 | 5.28 | | | |

| | | | | | | | | |
|---|---|---|---|---|---|---|---|---|
| NAN1 | 0.000044 | 2.72 | PIM1 | 0.000054 | 3.25 | | | |
| ERB1 | 0.000044 | 3.27 | C1_08110W | 0.000054 | 4.95 | | | |
| UBR1 | 0.000041 | 2.18 | C1_05630C | 0.000053 | 3.25 | | | |
| RPN2 | 0.000039 | 2.33 | MSN4 | 0.000052 | 2.25 | | | |
| DCK1 | 0.000039 | 2.61 | C7_03160W | 0.000052 | 2.15 | | | |
| C1_07340W | 0.000036 | 2.09 | VMA5 | 0.00005 | 3.87 | | | |
| PIM1 | 0.000034 | 2.63 | CR_03200C | 0.00005 | 1.8 | | | |
| C6_03460W | 0.000032 | 2.87 | RRP6 | 0.00005 | 5.73 | | | |
| RGA2 | 0.000031 | 1.77 | NUP82 | 0.000049 | 5.26 | | | |
| C1_11860W | 0.00003 | 1.11 | C2_04370W | 0.000048 | 3.41 | | | |
| C1_01590C | 0.00003 | 7.04 | SRP40 | 0.000046 | 6.78 | | | |
| PGA63 | 0.000029 | 1.49 | C1_03790C | 0.000046 | 4.93 | | | |
| SSD1 | 0.000029 | 1.8 | SEC12 | 0.000046 | 2.26 | | | |
| C1_08180C | 0.000028 | 2.44 | DNM1 | 0.000045 | 1.73 | | | |
| SPT6 | 0.000026 | 1.27 | C1_02240W | 0.000045 | 2.17 | | | |
| MLP1 | 0.00002 | 19.05 | PRO2 | 0.000044 | 3.59 | | | |
| GCN1 | 0.000015 | 0.9 | PMA1 | 0.000044 | 5.25 | | | |
| MDN1 | 0.000015 | 2.39 | C1_14080W | 0.000043 | 3.14 | | | |
| TRA1 | 0.00001 | 1.93 | SHM2 | 0.000041 | 2.98 | | | |
| | | | RPD3 | 0.000041 | 3.75 | | | |
| | | | C6_02370C | 0.00004 | 2.9 | | | |
| | | | C1_09040C | 0.00004 | 3.29 | | | |
| | | | PNG2 | 0.00004 | 1.83 | | | |
| | | | CAP1 | 0.000039 | 2.81 | | | |
| | | | C1_00060W | 0.000038 | 2.73 | | | |
| | | | SRV2 | 0.000036 | 2.39 | | | |
| | | | CRM1 | 0.000036 | 2.97 | | | |

| | | | | | | | | |
|---|---|---|---|---|---|---|---|---|
| | | | PEX1 | 0.000036 | 1.74 | | | |
| | | | RPA190 | 0.000035 | 2.58 | | | |
| | | | CCT6 | 0.000035 | 3.76 | | | |
| | | | C5_02380W | 0.000033 | 2.68 | | | |
| | | | PYC2 | 0.000033 | 2.63 | | | |
| | | | KRE30 | 0.000032 | 1.97 | | | |
| | | | C1_01590C | 0.000032 | 3.16 | | | |
| | | | C7_01030C | 0.000032 | 1.73 | | | |
| | | | MSI3 | 0.000028 | 3 | | | |
| | | | UTP8 | 0.000028 | 2.73 | | | |
| | | | C2_00360C | 0.000028 | 1.86 | | | |
| | | | CDC53 | 0.000026 | 1.86 | | | |
| | | | C4_00420C | 0.000024 | 2.47 | | | |
| | | | TOM1 | 0.000024 | 1.58 | | | |
| | | | ERB1 | 0.000023 | 2.24 | | | |
| | | | SNF2 | 0.000023 | 1.18 | | | |
| | | | NAB3 | 0.000023 | 2.39 | | | |
| | | | FAS2 | 0.000021 | 0.8 | | | |
| | | | SPO72 | 0.000021 | 0.7 | | | |
| | | | PFK1 | 0.00002 | 1.82 | | | |
| | | | CDC39 | 0.000019 | 0.69 | | | |
| | | | MET10 | 0.000018 | 1.46 | | | |
| | | | SSN6 | 0.000018 | 1.48 | | | |
| | | | MDN1 | 0.000016 | 1.33 | | | |
| | | | C2_02540W | 0.000011 | 0.93 | | | |

**Table S2: Output from Venn diagram containing the list of overlapping and non-overlapping proteins in the three immunopurifications.**

| Common elements in TAF12L-FLAG TBP-TAP TAF11-TAP : | Common elements in TBP-TAP TAF11-TAP : | Common elements in TAF12L-FLAG TBP-TAP : | Common elements in TAF12L-FLAG TAF11-TAP | TAF12L only | TBP only | TAF11 only |
|---|---|---|---|---|---|---|
| C1_10620W | TBP1 | RPL43A | NGG1 | NOP10 | C7_01400C | C2_03160C |
| GAR1 | C7_00340C | RPL25 | RPS19A | BBC1 | C4_04600C | RPC10 |
| RPL4B | RIM1 | RPL39 | RPS25B | ACS1 | TRI1 | CMD1 |
| RPL10A | CR_03460W | SSC1 | C4_04390W | ADA2 | CR_04310C | C7_02660C |
| RPL2 | TAF145 | RPL23A | YML6 | CR_10450C | TOA2 | TIF34 |
| RPP2A | TAF4 | RPL32 | C4_02260C | CR_04870C | BRF1 | CR_06800C |
| RPP0 | C2_02500W | RPS15 | C1_05270C | SPT7 | C4_05820W | MNT1 |
| RPL8B | TAF12 | ACT1 | TPI1 | GCN5 | CR_05150W | C4_04330C |
| RPL3 | C3_03930W | RPL28 | C5_00150C | NHP2 | MRPL19 | C1_01860W |
| CR_04450C | SIS1 | TRA1 | NOG1 | SPT20 | PTC2 | C7_00070C |
| RPP1A | C2_10680W | RPS18 | UTP9 | C2_05830C | C4_03090W | C5_02210W |
| RPL10 | HTA1 | RPL13 | C3_00450C | C7_00450C | RFA1 | SGT2 |
| RPL12 | TAF19 | CR_04240C | VMA8 | RPS3 | MCI4 | MP65 |
| SSB1 | TSM1 | RPL35 | CR_00490W | C5_02900W | DCW1 | LAB5 |
| RPS12 | SKP1 | RPL21A | UTP13 | C3_03100C | C1_09620C | C6_00290W |
| RPL15A | C4_04160W | RPS24 | RPA135 | TAF12L | C5_00560W | C3_03410C |
| RPS6A | KGD2 | RPL17B | DRG1 | RPS23A | C1_01680C | CCR4 |
| RPL18 | DBP5 | PR26 | C2_05300C | C6_02310W | C1_01370C | HEM14 |
| RPS8A | C7_00790W | C5_01700W | CAP1 | C3_07050W | C4_06730C | SSZ1 |
| TAF60 | KGD1 | RPL11 | SRV2 | C3_05790C | C7_01210C | MPP10 |
| RPL14 | C1_11200W | HTA2 | C2_00360C | CR_04110W | C4_05900C | NOT4 |

| | | | | | | |
|---|---|---|---|---|---|---|
| RPL24A | MGE1 | C2_07680W | | RPS21B | MAK16 | C4_03290W |
| SBP1 | SDH2 | RPS20 | | C1_11080W | GSP1 | C5_03550W |
| C2_05710C | HEM1 | C2_05410W | | RPS10 | CR_10740W_A | PIN4 |
| TAF10 | NSP1 | GFA1 | | RPS16A | C2_07370W | ROM2 |
| C5_03830C | C4_06680C | IMG2 | | TEF2 | DPM1 | C1_12030W |
| RPS14B | SEC24 | C1_02330C | | RPS28B | PRX1 | PAN1 |
| TEF1 | LSP1 | C5_01540W | | CR_04390C | RFA2 | C1_04290C |
| HHF22 | C6_02690C | RPL20B | | RPS5 | MGM101 | C1_00960C |
| C1_00180W | MDJ1 | RPT5 | | C4_03040W | ZUO1 | C1_06550W |
| RPL6 | MET3 | RPL5 | | C1_03620C | C6_00550W | C7_00570W |
| ERG13 | YWP1 | ATP1 | | C5_00820W | C1_03280W | C1_03290W |
| C1_00710C | AAF1 | RVS161 | | MRPL37 | CR_04170W | C7_04300W |
| C2_03560C | C6_00170C | CR_10350C_B | | RPT6 | CDC28 | C2_06170C |
| RPL19A | NUP49 | C5_01050C | | CR_10820W_A | C5_03980W | C4_01060W |
| RPL9B | C2_02170W | RPT4 | | AHP1 | AHA1 | C5_00190C |
| C3_04380C | C3_05880C | C5_04910W | | C6_02470W | C2_01390W | |
| RPL27A | RTG3 | C5_02660C | | UBP8 | C7_02960C_A | |
| RPL30 | C3_00130C | C2_03950W | | CR_07220C | GPD1 | |
| RVB1 | C5_04720C | RPN13 | | TSA1B | CR_10490W_A | |
| UBI3 | C3_00850C | SAM2 | | C6_03430C | ABP1 | |
| NOP5 | CR_02610C | MRPL40 | | C1_00110W | RPC53 | |
| C2_07190C | MIR1 | C2_01740C | | RPS17B | PHO23 | |
| MRT4 | TRP5 | RHR2 | | C3_06970W | DIP2 | |
| RPS1 | SRO77 | C3_01720C | | RSM22 | VID27 | |
| RPP2B | IDH2 | ALI1 | | RPT1 | FUN12 | |
| SIK1 | C2_03360W | GCD2 | | SRB1 | C3_07460W | |
| HSP70 | C1_06590C | C4_06210C | | C2_05160C | CTA7 | |

| | | | | | | |
|---|---|---|---|---|---|---|
| RPP1B | SLK19 | RPN1 | | C5_04630W | RAD52 | |
| TUB1 | FLO8 | C1_00590W | | RPT2 | RET2 | |
| VMA2 | CAM1 | CCT7 | | ACH1 | C1_13320C | |
| ASC1 | CR_08290W | PGI1 | | TRP99 | SDS24 | |
| TDH3 | ECM17 | CEF3 | | PFK26 | GZF3 | |
| MRP7 | MTS1 | RPL7 | | CR_07510W | ILS1 | |
| C1_04180W | SLA2 | CR_07320C | | PRE8 | C5_03000C | |
| ADH1 | MLS1 | NOP1 | | NOG2 | CR_05550C | |
| C1_00900W | PDI1 | C2_09660W | | C3_06760W | SEC18 | |
| TIF | C6_04290W | C1_14500C | | C1_11880W | RDH54 | |
| C1_05720W | UBR1 | UTP18 | | UBC4 | SMC1 | |
| BMH1 | C1_07340W | C1_10470W | | C2_02270C | CR_03220C | |
| RPS9B | C6_03460W | RPD31 | | RNH1 | MCM2 | |
| EFT2 | RGA2 | FBA1 | | GSY1 | CDC54 | |
| RPS21 | C1_11860W | CIC1 | | ALD5 | SEC21 | |
| RVB2 | SSD1 | C6_03380W | | C1_05650W | C2_04620W | |
| HSP60 | C1_08180C | NSA2 | | C7_03830C | C2_06980W | |
| C7_03000C | MLP1 | NOP15 | | C1_00040W | CR_10470C_B | |
| GPM1 | | RPN2 | | C7_03660C | ELF1 | |
| RPS7A | | UTP4 | | C5_03410C | C3_05360C | |
| C1_03370W | | YTM1 | | YST1 | C4_07060W | |
| PDC11 | | CR_10830C_A | | VMA6 | DCK1 | |
| C2_04120C | | CR_03120W | | C1_01580W | | |
| C4_03410W | | C5_04990W | | IDH1 | | |
| HSP90 | | RPC40 | | RPN12 | | |
| SSA2 | | C3_01850W | | SAR1 | | |
| CR_07080W | | CDC10 | | C1_01600W | | |

| | | | | | | |
|---|---|---|---|---|---|---|
| C1_12610W | | TCP1 | | THS1 | | |
| RPS42 | | GCN1 | | RNR21 | | |
| C5_00030W | | ARP7 | | GLN3 | | |
| CDC19 | | HOG1 | | C7_03840W | | |
| RNR1 | | HET1 | | CLC1 | | |
| RPS26A | | C5_00260W | | C6_02880W | | |
| ENO1 | | NSA1 | | ZCF29 | | |
| CR_00460C | | CSI2 | | PMM1 | | |
| YDJ1 | | PWP1 | | UTP5 | | |
| ADE4 | | C6_02350C | | C2_07220W | | |
| RPS13 | | SEC26 | | C3_04810C | | |
| SMT3 | | GLC7 | | CR_02420W | | |
| C4_05630W | | PIM1 | | CCJ1 | | |
| PDB1 | | VMA5 | | MRPL10 | | |
| ACS2 | | CR_03200C | | MYO2 | | |
| C1_12280C | | SRP40 | | DPS1-1 | | |
| KAR2 | | C1_03790C | | RAS1 | | |
| C1_00160C | | DNM1 | | C3_06700C | | |
| PGK1 | | C1_02240W | | C5_00080C | | |
| C2_04570W | | PRO2 | | C2_07290W | | |
| PRT1 | | PMA1 | | HXK2 | | |
| TKL1 | | RPD3 | | C3_07420W | | |
| LAT1 | | C6_02370C | | MSS116 | | |
| NIP1 | | C1_09040C | | MRPS9 | | |
| ARO8 | | PNG2 | | CSH1 | | |
| C4_04820C | | C1_00060W | | GRP2 | | |
| TAF14 | | RPA190 | | ARC35 | | |

| | | | | | | |
|---|---|---|---|---|---|---|
| LYS22 | | MSI3 | | NMD3 | | |
| HSP21 | | UTP8 | | C5_00790C | | |
| MRPL3 | | TOM1 | | CHA1 | | |
| PIL1 | | ERB1 | | KRR1 | | |
| PDX1 | | FAS2 | | LYS21 | | |
| HIS7 | | PFK1 | | AGO1 | | |
| FAS1 | | MET10 | | C6_04100W | | |
| SER33 | | MDN1 | | C5_04640C | | |
| URA2 | | C2_02540W | | ERG6 | | |
| SDH12 | | | | RNR22 | | |
| SUB2 | | | | LMO1 | | |
| PES1 | | | | SWD1 | | |
| PET9 | | | | SES1 | | |
| RPG1A | | | | C1_12240C | | |
| SEC27 | | | | MIS11 | | |
| PFK2 | | | | C6_03440W | | |
| CDC48 | | | | ARO9 | | |
| C3_02350W | | | | AIP2 | | |
| ATP2 | | | | HOM3 | | |
| ILV2 | | | | PBS2 | | |
| CHC1 | | | | GAD1 | | |
| PDA1 | | | | HAS1 | | |
| C1_00930C | | | | GGA2 | | |
| GLT1 | | | | APE2 | | |
| TIF4631 | | | | C1_00640C | | |
| HSP104 | | | | C2_06200C | | |
| SPT6 | | | | ROB1 | | |

| | | | | | | |
|---|---|---|---|---|---|---|
| SNF12 | | | | RGD1 | | |
| LPD1 | | | | VPS1 | | |
| NAN1 | | | | C1_08110W | | |
| PGA63 | | | | MSN4 | | |
| ACC1 | | | | C7_03160W | | |
| DED1 | | | | RRP6 | | |
| PPH21 | | | | NUP82 | | |
| C1_05630C | | | | SEC12 | | |
| C2_04370W | | | | C1_14080W | | |
| SHM2 | | | | CRM1 | | |
| C5_02380W | | | | PEX1 | | |
| KRE30 | | | | CCT6 | | |
| C1_01590C | | | | PYC2 | | |
| CDC53 | | | | C7_01030C | | |
| C4_00420C | | | | SNF2 | | |
| | | | | NAB3 | | |
| | | | | SPO72 | | |
| | | | | CDC39 | | |
| | | | | SSN6 | | |

**Table S3. List of Strains**

| Strain | RELEVANT GENOTYPE | SOURCE |
|---|---|---|
| SN95 | *arg4Δ/arg4Δ his1Δ/his1Δ URA3/ura3Δ::imm*$^{434}$ *IRO1/iro1Δ::imm*$^{434}$ | (Noble and Johnson, 2005) |
| SN87 | *ura3Δ-iro1Δ::imm434/URA3-IRO1, his1Δ/his1Δ, leu2Δ/leu2Δ* | (Noble and Johnson, 2005) |
| ISC11 | SN95 *HAH1-*$P_{MAL2}$*-TAF12L/HIS1-*$P_{MAL2}$*-TAF12L* | (Sinha et al., 2017) |
| ISC12 | SN95 *HAH1-*$P_{MAL2}$*-TAF12/HIS1-*$P_{MAL2}$*-TAF12* | (Sinha et al., 2017) |
| ISC33 | SN87 *TBP::TAP-C.d.HIS1/TBP::TAP-C.m.LEU2 TAF12L::*$HA_3$*-FRT/TAF12L::*$HA_3$*-SAT1 FLP* | (Sinha et al., 2017) |
| ISC49 | ISC44 *TAF11::TAP-C.d.HIS1/TAF11::TAP-C.m.LEU2* | (Sinha et al., 2017) |
| SKC3 | SN87 *TAF12L::*$His_6$*-*$Gly_2$*-*$FLAG_3$*-FRT/TAF12 L::*$His_6$*-*$Gly_2$*-*$FLAG_3$*-SAT1-FLP* | (Sinha et al., 2017) |
| SKC6 | SN87 *TAF12:: *$His_6$*-*$Gly_2$*-*$FLAG_3$*-FRT/TAF12::*$His_6$*-*$Gly_2$*-*$FLAG_3$*-SAT1-FLP* | (Sinha et al., 2017) |
| SKC8 | SN87 *ADA1::*$His_6$*-*$Gly_2$*-*$FLAG_3$*-FRT/ADA1* | (Sinha et al., 2017) |
| SKC12 | SN87 *TAF4::*$His_6$*-*$Gly_2$*-*$FLAG_3$*-FRT/TAF4::*$His_6$*-*$Gly_2$*-*$FLAG_3$*-SAT1-FLP* | (Sinha et al., 2017) |
| VBC3 | *SN95* $P_{MAL2}$*TAF4/TAF4* | This work |
| VBC4 | *SN95* $P_{MAL2}$*TAF4/*$P_{MAL2}$*TAF4* | This work |
| VBC5 | *SN95* $P_{MAL2}$*ADA1/PADA1* | This work |
| VBC6 | *SN95* $P_{MAL2}$*ADA1/*$P_{MAL2}$*ADA1* | This work |

**Table S4. List of Oligonucleotides**

| OLIGO NUMBER | LENGTH | SEQUENCE (5' to 3') | NOTES |
|---|---|---|---|
| ONC114 | 18 | 5'-GGTGCCACTGATCCATTG-3' | Position 61 to 78 within *CaARG4* ORF |
| ONC115 | 18 | 5'-GCCAACATATCCATAGTTAAAGC-3' | Position 1108 to 1130(c) within CaARG4 ORF |
| ONC116 | 21 | 5'-CCGTATTCCATGATTGCTATG-3' | Diagnostic primer within CaMAL2 promoter; position -230 to -210 upstream of target genes ATG. |
| ONC104 | 18 | 5'-TAAAAATATCGCACTCAC-3' | 3'-primer for *ADH1;* +1374 to +1356 wrt ATG |
| ONC114 | 18 | 5'-GGTGCCACTGATCCATTG-3' | Position 61 to 78 within Ca*ARG4ORF* (Ca*ARG4*-F61) |
| ONC115 | 23 | 5'-GCCAACATATCCATAGTTAAAGC-3' | Position 1108 to 1130(c) within *CaARG4ORF* (Ca*ARG4*-R1130) |
| ONC123 | 18 | 5'-GCCCTTCTGCCTGGAGTA-3' | Diagnostic PCR primer within the non-repeat sequence of *HIS1* of pHAH |
| ONC 400 | 25 | 5'-CAGAGAAAGCGGAGGAAAATAGTAA-3' | Forward RT primer for TAF12 |
| ONC 401 | 25 | 5'-ATAATGGTTTGGGTTTCGACTTAGA-3' | Reverse RT primer for TAF12 |
| ONC 431 | 23 | 5'-AGTCGAAAGAAAATTGGCTGCTA-3' | Forward RT primer for RPS8A |
| ONC 432 | 24 | 5'-CAGAACCGAATTGAGAGTCAACAG-3' | Reverse RT primer for RPS8A |
| ONC 439 | 21 | 5'-GGGCTGCAGCATTACTTTTAG-3' | Forward RT primer for ADA1 |
| ONC 440 | 21 | 5'-GCCGTCGAATCTGTTTTGTTT-3' | Reverse RT primer for ADA1 |
| ONC 520 | 21 | 5'-CAACTGGACGCTCAGGAACTC-3' | Forward RT primer for TAF12L |

| | | | |
|---|---|---|---|
| ONC 521 | 20 | 5'-GACGTGTAATGCCAGCCAAA-3' | Reverse RT primer for TAF12L |
| ONC 616 | 21 | 5'- GCCTGAACCGACGCAAACTTG -3' | Upcheck Forward primer ADA1 for screening |
| ONC 617 | 25 | 5'- GATTGCTCAAGAATTGATCCAGACC -3' | DownCheck Reverse primer for ADA1 for screening |
| ONC 667 | 21 | 5'- CTCATCAAAGGACTCGGGAAA-3' | Forward primer specific for CaADA1 ORF |
| ONC 1060 | 76 | 5'TTAAGGAAAACTCAATTATTAACAATCTTGAAATCACCGAATTATATCACATAAATCCCTTCGTACGCTGCAGGTC 3' | Forward long primer for TAF4 promoter replacement through HAH |
| ONC 1061 | 88 | 5'GGAGTCCTCACTGTTTTCTAATTGTCTCTTCAAATTAGAGGATTCTTGAGGTGTACTTGTCATTGTAGTTGATTATTAGTTAAACCAC3' | Reverse long primer for TAF4 promoter replacement through HAH |
| ONC 1062 | 78 | 5'TTCCCCCCAATAATACCTTTTATTTTGGCAATTTACATTTGAGTTACTATTTCTTTGATTCTTCGTACGCTGCAGGTC3' | Forward long primer for ADA1 promoter replacement through HAH |
| ONC 1063 | 85 | 5'TCCATTTTTCAAAGGATTGATAGTAGTTGTAGAAGATCCATCAGCGATTTGAGATGTCATTGTAGTTGATTATTAGTTAAACCAC 3' | Reverse long primer for ADA1 promoter replacement through HAH |
| ONC 1294 | 20 | 5'-TTTGGAAGCAACACTGGACA-3' | Downcheck Reverse primer for TAF4 |
| ONC1300 | 22 | 5'-GACTATGAATCCCGGGAGAAAG-3' | Forward RT primer for TAF4 |
| ONC 1301 | 21 | 5'-TTTCCCGAGTCCTTTGATGAG -3' | Reverse RT primer for TAF4 |

## Experimental procedures

### *Strains and growth conditions*

*C. albicans* strains SN87 and SN95 were used as parental strains. All strains were cultured in yeast extract-peptone (YP) rich medium with either glucose or maltose as carbon source, as indicated. The *C. albicans* genome sequence data and annotations were obtained from the Candida Genome Database (CGD). Details of strain construction are described in the supplemental text. The list of strains used are provided in Table S1, and the oligonucleotides used in Table S2.

### *Immunoblotting*

Indicated strains were cultured in YPM at 30°C for 16-18h with shaking at 220 rpm, and diluted the culture into fresh YPD and collected cells at 0, 2, 4, and 6h by centrifugation. Whole-cell extracts were made by bead beating using chilled glass beads for 8 cycles in lysis buffer as described (36), and total protein estimated using Bradford assay (Bio-Rad) using BSA as a standard. Cell extracts containing ~100µg total protein was loaded into each well of 8% SDS-PAGE, blotted to Protran nitrocellulose membrane (GE Healthcare), probed with 1:1000 dilution of anti-TAF12 and TAF12L rabbit polyclonal antibodies, or TAF4 and Ada1 mouse polyclonal antibodies, as described previously (19). The blots were further incubated with 1:20000 dilution of goat anti-rabbit or anti-mouse HRP conjugate secondary antibodies, and the blots were washed and exposed to Hyperfilm ECL X-ray film (GE Healthcare) and visualized using the ECL Plus chemiluminescent system. The images were scanned, edited in Adobe Photoshop and combined using Adobe Illustrator software.

### *RNA extraction and RT-PCR*

*C. albicans* strains were grown as described under Immunoblotting. About 10 $OD_{600}$ were harvested by rapid filtration, and RNA was extracted using the hot-phenol method (37). One

microgram of RNA was subjected to DNase I treatment, and one-third was converted to cDNA using Superscript III cDNA synthesis kit using random hexamer primers. RT-qPCR was performed with 4µl of 1:50 diluted cDNA as template and gene-specific primers (Table S2) in duplicate for each sample. The relative mRNA levels were calculated by ΔCq (WT)-ΔCq (mutant), where ΔCq=Cq(gene)-Cq (*SCR1*), and ΔΔCq values were plotted using GraphPad Prism 8.0 as relative mRNA levels (Mutant/WT). The error bars indicate ± SD (N = 3).

### *Polysome extract preparation and polysome analysis*

*C. albicans* strain SN95 was cultured overnight till saturation in YPD medium, diluted to fresh YPD, grown till $OD_{600}$ ~0.6-0.8. 1mg/ml CHX was added to the culture for 5 min, and cells from 100 ml were harvested, typically total ~70 $OD_{600}$. The cell pellets were washed with lysis buffer (20 mM HEPES KOH pH 7.5, 150 mM KCl, 10 mM $MgCl_2$, 0.1% (vol/vol) NP-40, and 1x Roche Complete protease inhibitor) containing with or without 1mg/ml CHX, resuspended in ~200µl of the same buffer with or without CHX and lysed by glass bead lysis for 10 min each using Vortex Genie instrument. Lysate was cleared by spinning at 10,000xg for 10 min at 4°C. Cell extracts containing 5.0 A260 units were loaded onto a 12ml 10-50% (wt/vol) sucrose gradient made in buffer containing 10mM Tris Acetate pH7.4, 70mM Ammonium Acetate, 4mM Magnesium Acetate and centrifuged in a SW41Ti rotor at 35000 rpm (194,000 ×g) for 160 min at 4°C in Beckman Coulter ultracentrifuge. At the end of the centrifugation, samples were fractionated using an automated gradient maker and fractionator (BioComp Gradient Master and Fractionator, USA), and A260nm of the fractions were recorded and plotted.

### ***RNA-Immunoprecipitation***

The polysome extracts were prepared as described above except that the cell pellets were washed with 10 ml lysis buffer containing either 1mg/ml CHX (+CHX culture) or 1mg/ml puromycin (+Puro culture), and cells again resuspended in ~600µl of the lysis buffers with

CHX or Puro, and 40U RNase inhibitor (Thermo Fischer) was added to each sample just prior to the extract preparation. Cells were lysed in presence of glass beads and vortexed for 10 Min, and ~4mg equivalent total proteins from whole cell extracts were used for immunoprecipitation using 30µl anti-FLAG M2 affinity gel (Sigma/Merck) for 4h at 4°C. The beads were then washed four times with the washing buffer (lysis buffer with 350mM KCl), and beads resuspended in AE buffer (50 mM NaOAc, 10 mM EDTA) with 20µl 10% SDS per sample, extracted with an equal volume of pre-warmed phenol, followed by chloroform extraction, and the aqueous phase was collected, and ethanol precipitated in presence of NaOAc and 1µl glycogen. The pellet was washed, dried, and dissolved in 10µl nuclease-free water (Ambion). Ten percent of the input cell extract from each sample was used for RNA preparation as input RNA control as above. Five-microliters of 1:10 diluted input RNA and 5 µl of immunoprecipitated RNA were treated with DNase I and used for cDNA preparation, and qPCR was carried out as described above. Quantitative enrichment analysis was carried out using the formula $100 \times 2^{[(Cq(Input) - 3.322) - Cq(IP)]}$ and expressed as % Input RNA as described previously (35).

### *Affinity Purification and LC-MS/MS analysis*

*C. albicans* TAF12L-FLAG (SKC3), TBP-TAP (ISC33), TAF11-TAP (ISC49), and control (SN87) strains were precultured for 14h in YPD and then diluted into six litres of YPD and grown till OD600 ~2-3 and processed as follows. Cells were harvested at 7000rpm for 20min at 4°C in Beckman JLA-8.1 rotor, cell pellets washed with 30ml H350 buffer, and resuspended in either 20ml H350 buffer (25mM HEPES-KOH, pH=7.5, 350mM KCl, 2mM MgCl2, 1mM EDTA, 10% glycerol, 0.02% NP40) for FLAG samples, or 12ml TAP extraction buffer (40 mM HEPES-KOH, pH 7.5, 10% Glycerol, 350mM NaCl, 0.1% Tween-20) with protease inhibitors (1mM PMSF, 1µg/ml pepstatin A, 2µg/ml leupeptin, and 100µl Sigma Yeast Protease

Inhibitor (P8250) per 50ml cell suspension). The cells were lysed using 0.5mm glass beads in a BeadBeater (Biospec Products) for 15 min with 30 sec On and 1.5 min Off cycles, with change of ice in the external chamber at 4°C, and heparin (0.5mg for FLAG extracts, or 1mg for TAP extracts) and 125 units benzonase were added, centrifuged at 45000 rpm for 1.5h in a 70Ti rotor in a Beckman ultracentrifuge, and the supernatants were collected. For FLAG purification, 400µl pre-washed anti-FLAG M2 affinity agarose gel (A2220, Sigma) was added to the SKC3 and SN87 lysates, and incubated with rotation at 4°C for 3-5h. The beads were washed, and bound proteins were eluted by the addition of 250µg/ml 3xFLAG peptide (F4799, Sigma), incubated for 30 min at 4°C, and elution repeated 3-4 times and fractions collected.

For purification of TAP-tagged complexes, two-step purification was employed as outlined briefly. The lysates from TAP-tagged (ISC33 and ISC49) and untagged control (SN87) strains were incubated with 400µl IgG-Sepharose 6 Fast flow (GE healthcare) that was pre-washed with TAP extraction buffer and incubated for 2h on a rotator. The beads were collected by gentle centrifugation at 200-150 xg -, and the supernatant was separated. The bead fractions were washed five times with TAP extraction buffer at 4°C, and bound proteins were eluted by TEV cleavage as follows. The beads were incubated with 1ml TEV cleavage buffer containing 10µl AcTEV (Invitrogen), incubated with rotation at 4°C for 16h, and the eluates were collected. Next, the eluate was added to 250µl pre-washed Calmodulin-Sepharose beads in calmodulin binding buffer (CBB; 10mM Tris, pH 8.0, 1mM MgOAc, 1mM Imidazole, 2mM $CaCl_2$, 0.1% NP-40, 10% Glycerol, 0.3M NaCl and protease inhibitors) containing 1M $CaCl_2$ and incubated at 4°C for 3h on a rotator, and the beads washed five times with CBB (0.15M NaCl). The bound proteins were eluted with 200µl calmodulin elution buffer (10mM Tris, pH 8.0, 150mM NaCl, 1mM MgOAc, 1mM imidazole, 2mM EGTA, 0.1% NP-40, 10% Glycerol, protease inhibitors), serially seven times, and the fractions were collected. The eluted samples

were precipitated using 20% (w/v) TCA, washed with cold acetone, air-dried and submitted for LC-MS/MS mass spectrometry.

**Multidimensional Protein Identification Technology (MudPIT)**

TCA-precipitated protein pellets were resuspended in 30 µl of 100mM Tris-HCl, pH 8.5, 8 M urea, reduced with 5mM TCEP (Tris(2-Carboxylethyl)-Phosphine Hydrochloride, Pierce), and alkylated with 10 mM IAM (Iodoacetamide, Sigma). As described (38), a two-step digestion procedure was used. Endoproteinase Lys-C (Roche) was added to 0.5 µg for at least 6h at 37°C, then the sample was diluted to 2M urea with 100mM Tris-HCl, pH 8.5. Calcium chloride was added to 2mM and the digestion with trypsin (0.5 µg) was let to proceed overnight at 37°C while shaking. The reaction was quenched by adding formic acid to 5% and the peptide mixture was loaded onto a 100µm fused silica (FS) microcapillary column packed with 8 cm of reverse phase material (Aqua, Phenomenex) and connected to a 250µm FS column packed with 3 cm of 5-µm Strong Cation Exchange material (Partisphere SCX, Whatman), followed by 2 cm of 5-µm $C_{18}$ reverse phase(39).

The loaded microcapillary column was placed in-line with a Quaternary Agilent 1100 series HPLC pump. Overflow tubing was used to decrease the flow rate from 0.1 ml/min to about 200–300 nl/min. Fully automated 10-step chromatography runs were carried out (40). Three different elution buffers were used: 5% acetonitrile, 0.1% formic acid (Buffer A); 80% acetonitrile, 0.1% formic acid (Buffer B); and 0.5M ammonium acetate, 5% acetonitrile, 0.1% formic acid (Buffer C). Peptides were sequentially eluted from the SCX resin to the reverse phase resin by increasing salt steps, followed by an organic gradient. The last two chromatography steps consisted a high salt wash with 100% Buffer C followed by the acetonitrile gradient. The application of a 2.5 kV distal voltage electrosprayed the eluting peptides directly into a LTQ linear ion trap mass spectrometer equipped with a nano-LC

electrospray ionization source (Thermo, San Jose, CA). Full MS spectra were recorded on the peptides over a 400 to 1,600 *m*/*z* range, followed by five tandem mass (MS/MS) events sequentially generated in a data-dependent manner on the most intense ions selected from the full MS spectrum (at 35% collision energy). Mass spectrometer scan functions and HPLC solvent gradients were controlled by the Xcalibur data system (Thermo Scientific, San Jose, CA). SEQUEST (41) was used to match MS/MS spectra to peptides consisting of 9269 non-redundant proteins derived from all ORF translation of *Candida albicans* SC5314 Assembly 22 (20), as described (42). Usual contaminants (such as human keratins, IgGs, and proteolytic enzymes) were removed. To estimate false discovery rates, 9623 randomized amino acid sequences derived from each non-redundant protein entry was used. The validity of peptide/spectrum matches was assessed using the SEQUEST-defined parameters, cross-correlation score (XCorr) and normalized difference in cross-correlation scores (DeltCn). Spectra/peptide matches were only retained if they had a DeltCn of at least 0.08 and, minimum XCorr of 1.8 for singly-, 2.0 for doubly-, and 3.0 for triply-charged spectra. In addition, the peptides had to be fully-tryptic and at least 7 amino acids long. Combining all runs, proteins had to be detected by at least 2 such peptides, or 1 peptide with 2 independent spectra. Under these criteria, the final FDRs at the protein and peptide levels were less than 1%. DTASelect (39) was used to select and sort peptide/spectrum matches passing this criteria set. Peptide hits from multiple runs were compared using CONTRAST (39). To estimate relative protein levels, spectral counts were normalized as distributed Normalized Spectral Abundance Factors (dNSAFs) as described (43).

**Supplementary References**


37. Singh, R. P., Prasad, H. K., Sinha, I., Agarwal, N., and Natarajan, K. (2011) Cap2-HAP Complex Is a Critical Transcriptional Regulator That Has Dual but Contrasting Roles in Regulation of Iron Homeostasis in Candida albicans. *J. Biol. Chem.* **286**, 25154–25170
38. Green, M. R., and Sambrook, J. (2021) Total RNA Extraction from Saccharomyces cerevisiae Using Hot Acid Phenol. *Cold Spring Harb. Protoc.* **2021**, pdb.prot101691

39. Washburn, M. P., Wolters, D., and Yates, J. R. (2001) Large-scale analysis of the yeast proteome by multidimensional protein identification technology. *Nat. Biotechnol.* **19**, 242–247
40. McDonald, W. H., Ohi, R., Miyamoto, D. T., Mitchison, T. J., and Yates, J. R. (2002) Comparison of three directly coupled HPLC MS/MS strategies for identification of proteins from complex mixtures: single-dimension LC-MS/MS, 2-phase MudPIT, and 3-phase MudPIT. *Int. J. Mass Spectrom.* **219**, 245–251
41. Florens, L., and Washburn, M. P. (2006) Proteomic analysis by multidimensional protein identification technology. *Methods Mol. Biol. Clifton NJ.* **328**, 159–175
42. Eng, J. K., McCormack, A. L., and Yates, J. R. (1994) An approach to correlate tandem mass spectral data of peptides with amino acid sequences in a protein database. *J. Am. Soc. Mass Spectrom.* **5**, 976–989
43. Muzzey, D., Schwartz, K., Weissman, J. S., and Sherlock, G. (2013) Assembly of a phased diploid Candida albicansgenome facilitates allele-specific measurements and provides a simple model for repeat and indel structure. *Genome Biol.* **14**, R97
44. Zhang, Y., Wen, Z., Washburn, M. P., and Florens, L. (2010) Refinements to Label Free Proteome Quantitation: How to Deal with Peptides Shared by Multiple Proteins. *Anal. Chem.* **82**, 2272–2281

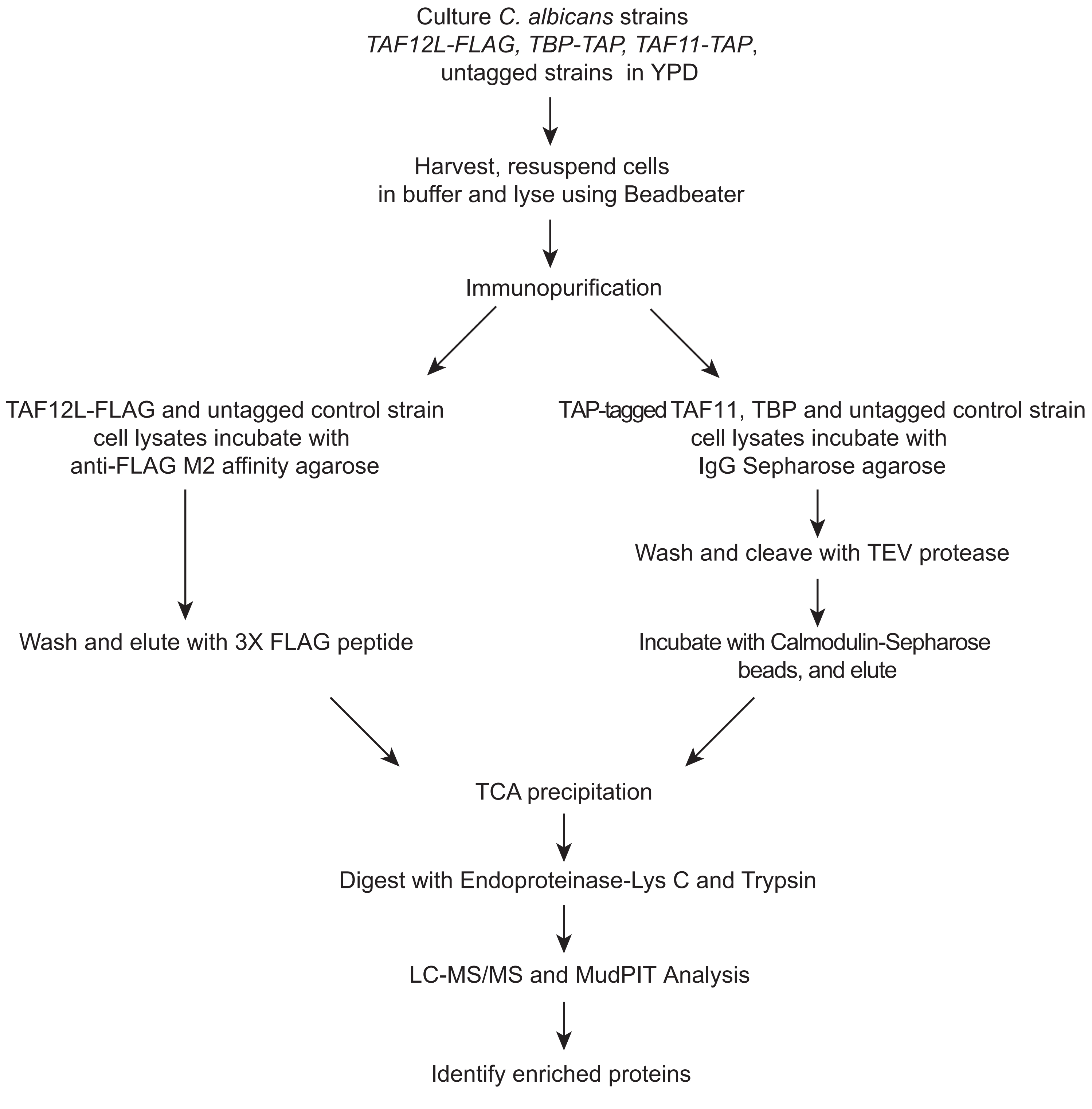


Fig. S1. Experimental scheme used to identify TFIID and SAGA subunits and the intertacing proteins using affinity purification, LC-MS/MS and MudPIT analyses

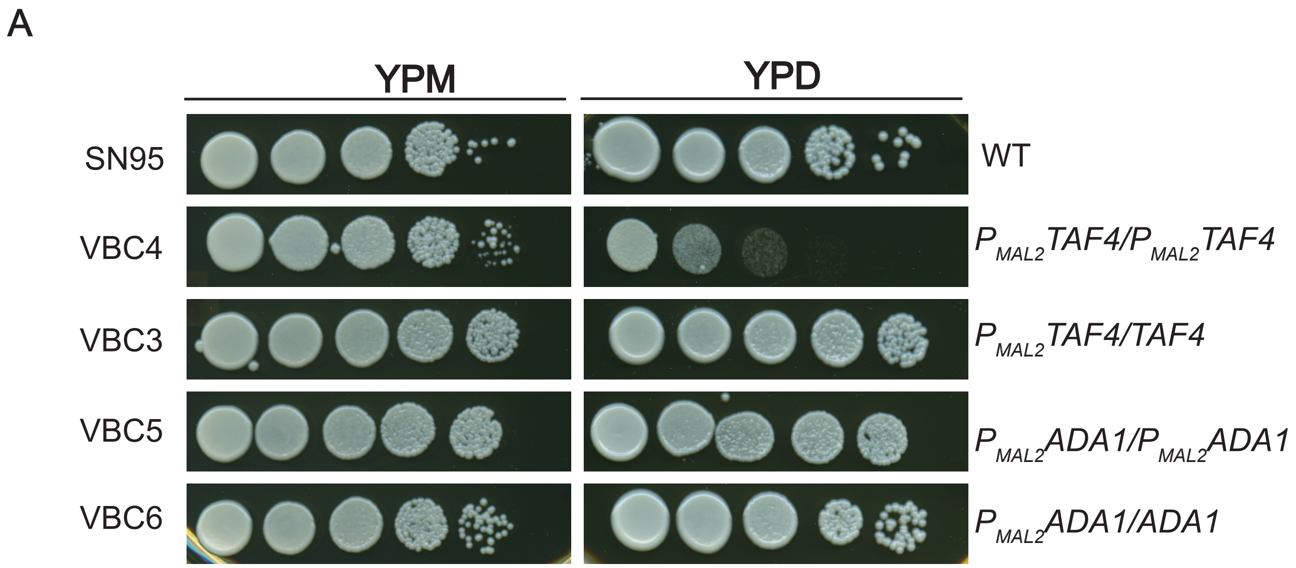


Figure S2. Growth phenotype analysis of TAF4 and ADA1 depleted strains. Growth phenotype of TAF4 and ADA1 depleted strains. Strains were grown in YPM till saturation and serially diluted, spotted on YPM and YPD plates, and incubated at 30°C. Plates were imaged at 36 h.

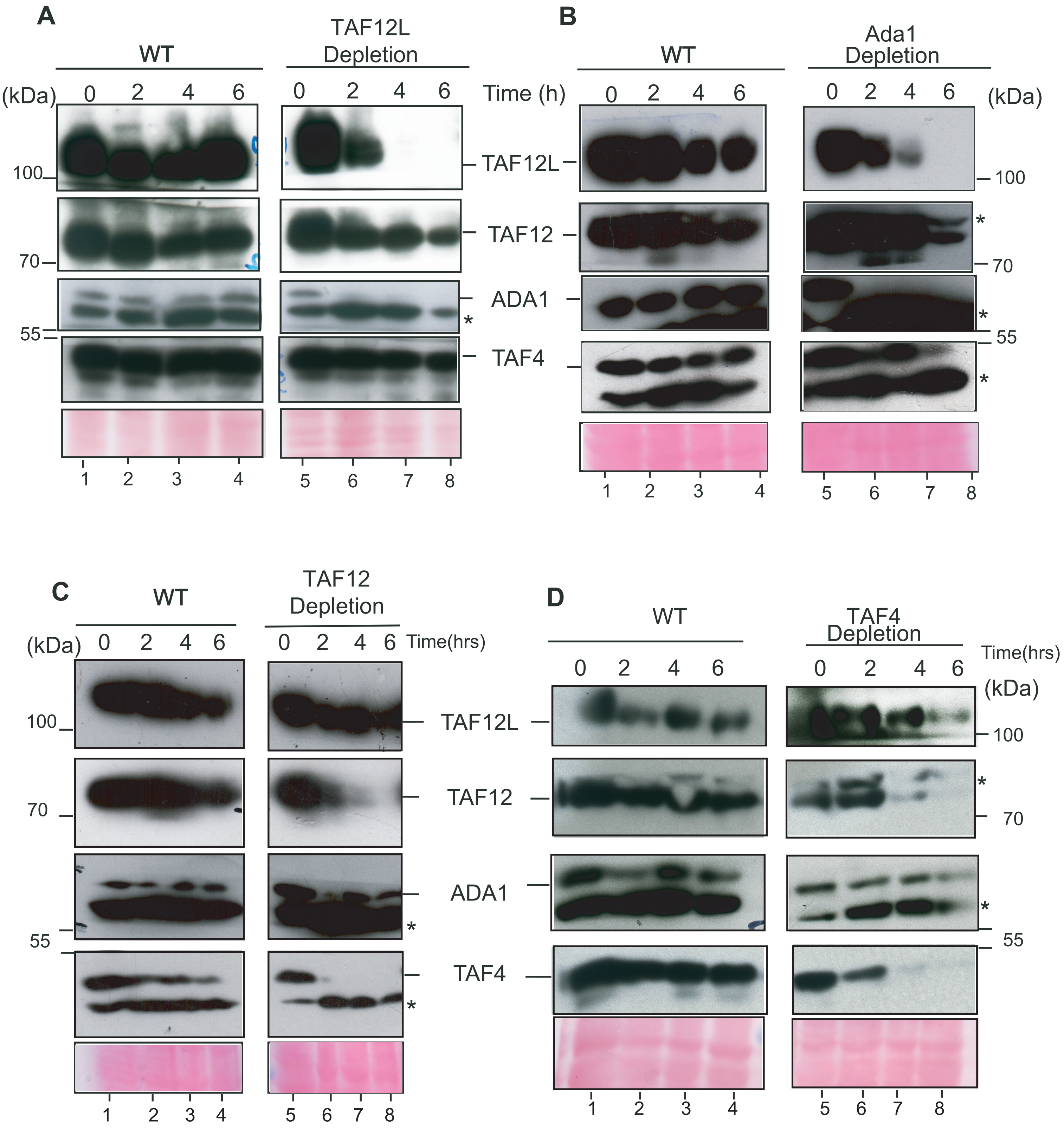


Fig. S3. (*A-B*) TAF12L and Ada1 depletion does not affect TAF12 or TAF4 protein abundance. (*C-D*), TAF12 and TAF4 depletion does not affect TAF12L or ADA1 protein abundance. Western blot analysis of cell extracts from ISC11 upon TAF12L depletion (*A*), VBC6 upon Ada depletion (*B*), ISC12 upon TAF12L depletion (*C*), VBC4 upon TAF4 depletion (*D*) or from control SN95 (WT) at indicated time points from 0h to 6h as in Figure 2.

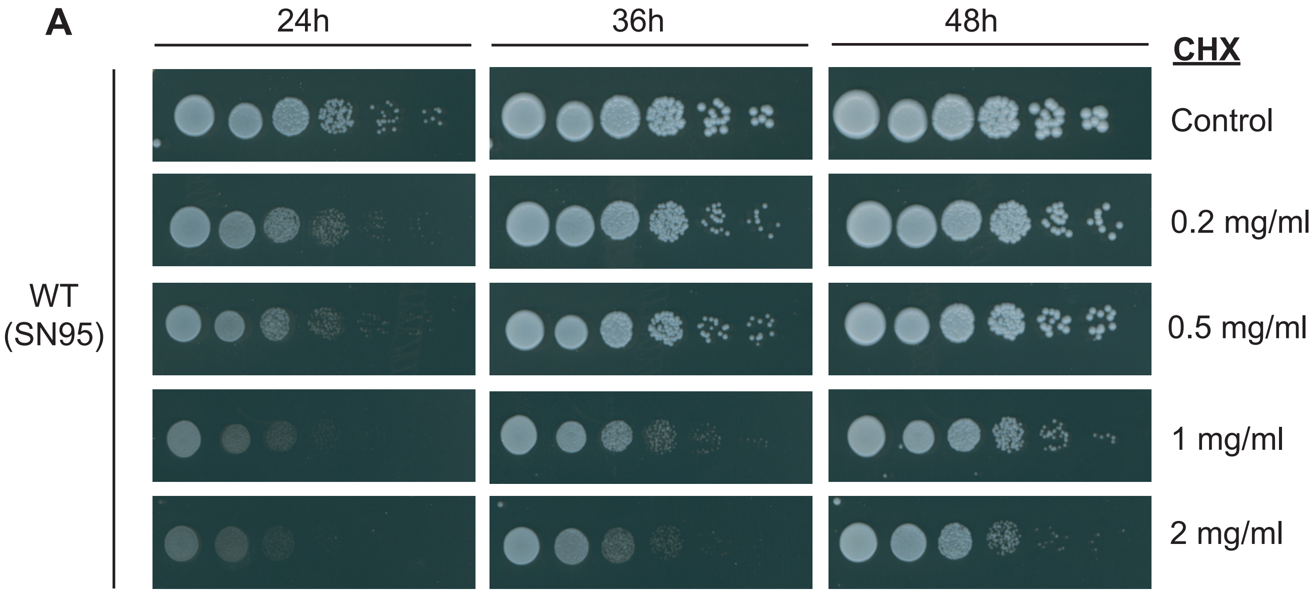


Fig. S4. Optimization of cycloheximide (CHX) concentration in C. albicans. WT strain (SN95) was grown in YPD for 16-18h, serially diluted and spotted on YPD, or YPD containing 0.2mg/ml, 0.5mg/ml, 1mg/ml, or 2mg/ml cycloheximide, and incubated at 30°C and photographed at indicated times.
